# The P-type calcium pump Spf1 regulates immune response by maintenance of the endoplasmic reticulum-plasma membrane contacts during *Candida albicans* systemic infection

**DOI:** 10.1080/21501203.2024.2409299

**Published:** 2024-11-07

**Authors:** Yuchao Ji, Dou Chen, Menglin Shao, Zhuo Liu, Mingchun Li, Qilin Yu

**Affiliations:** National Key Laboratory of Intelligent Tracking and Forecasting for Infectious Diseases, College of Life Sciences, Nankai University, Tianjin, China

**Keywords:** *Candida albicans*, P-type ATPase, endoplasmic reticulum-plasma membrane contact, immune response, virulence factor

## Abstract

Spf1 is an important P-type ATPase in *Candida albicans*, which functions as an endoplasmic reticulum calcium pump to maintain calcium homoeostasis. The deficiency of Spf1 attenuates the virulence of *C. albicans*. However, its impact on immune response remains to be investigated. This study discovered that deletion of *SPF1* resulted in a reduction of endoplasmic reticulum-plasma membrane contacts, an important structure mediating material and information exchange. This effect was attributed to the reduced plasma membrane localisation of the crucial endoplasmic reticulum-plasma membrane tethering proteins Ist2 and Tcb1/3. The reduction of the contacts led to a decrease in secretion of the virulence factors phospholipase, secreted aspartyl protease (SAP), candidalysin, and the cell wall-anchored protein Hwp1 during infection. Immunofluorescence staining and quantitative PCR assays further showed that the *SPF1* deletion led to a remarkable decrease in the levels of pro-inflammatory cytokines, suggesting the alleviation of the fungus-induced inflammatory response. Ultimately, the regulatory role of Spf1 in immune response significantly weakened the infectivity of *C. albicans*, and increased the survival rate of the hosts. This finding elucidated the role of fungal calcium pump-governed endoplasmic reticulum-plasma membrane contacts in regulation of immune response. It also makes it possible to regulate the host’s immune response via control of *SPF1* expression and functions, providing a theoretical basis for treating fungal infections.

## 1. Introduction

The human body is exposed to a complex environment, filled with various bacteria, fungi, and viruses both internally and externally. In this environment, it is crucial to maintain the stability of bodies, which requires effective immune responses and surveillance mechanisms. *Candida albicans* is a common fungus found in areas such as the oral cavity, digestive tract, and reproductive tract (Bai 2014; Talapko et al. 2021; Hu et al. 2024). Usually, the immune system can control the number of *C. albicans* to prevent infection. However, when the immune system is compromised or suppressed, the monitoring of fungal growth is weakened, leading to uncontrolled fungal growth and life-threatening infection. The mortality rate of this infection is about 40% in patients with immunodeficiency and receiving immunosuppressive therapy (Richardson and Moyes 2015). When *C. albicans* invades human tissue and causes infection, the body activates immune response. Immune cells such as neutrophils, monocytes, and macrophages are attracted and accumulate at the site of infection, triggering an inflammatory response (Bojang et al. 2021). The cytokines released by immune cells activate and recruit more immune cells, thereby clearing *C. albicans* in the infected area. For example, IL-1β is a major pro-inflammatory cytokine that can trigger and amplify inflammatory responses, activating the recruitment of various immune cells, and promoting the proliferation and differentiation of T and B cells (Griffiths et al. 2021). Similarly, IL-4 can stimulate activated T and B cells, induce macrophages to differentiate into M2-type macrophages, exert anti-inflammatory and repair functions, promote tissue repair, and regulate immune responses (Keegan et al. 2021). After B cell activation, it can produce pro-inflammatory cytokines IL-6, TNF-α, or anti-inflammatory cytokine IL-10, thereby affecting other immune cells (Harris et al. 2000; Duddy et al. 2004; Kulkarni et al. 2016). Interferon IFN-γ, produced by NK cells and T cells, can enhance the ability of macrophages and neutrophils to kill *C. albicans* (Trinchieri and Perussia 1985; Balish et al. 1998). TGF-β1 is an endogenous balancing factor used to maintain immune system balance and coordinate complex tissue repair processes, including its inhibitory effects on T and B cells, after organ damage or infection (Lodyga and Hinz 2020). In fungal infections, these immune cells and cytokines indicate that the strength of the normal immune system’s response to some extent represents the intensity of the infection, and also plays an important role in affecting mortality. Therefore, exploring the regulatory effects of different drugs and sites of action on the immune response in the process of *C. albicans* infection has become an important research direction for the treatment of candidiasis.

In eukaryotes, although calcium ion is one of the crucial second messengers in intracellular signalling pathways, it must be maintained at low levels in cells to avoid toxicity. Maintenance of calcium homoeostasis relies on several important intracellular active transport calcium pumps and transporters (Luna-Tapia et al. 2019). These include the calcium pump Spf1 on the endoplasmic reticulum, the calcium pump Pmr1 on the Golgi apparatus, the calcium transporter Cch1-Mid1 on the cell membrane, the calcium ion ATPase Pmc1 on the vacuole, the Ca^2+^/H^+^ exchanger Vcx1, etc. These proteins interact to maintain stable calcium ion concentrations inside and outside fungal cells and between organelles (Förster and Kane 2000; Yu et al. 2012a; Jiang et al. 2018). Among these calcium pumps, the Spf1 protein encoded by the *SPF1* gene is very important. Spf1 is a P-type ATPase located on the endoplasmic reticulum of *C. albicans*, responsible for maintaining calcium ion transport in the endoplasmic reticulum. Its absence stimulates calcium influx and leads to an increase in cellular calcium concentration (Cronin et al. 2002). Moreover, Spf1 also plays an important role in the development of hyphae, and its absence leads to an overall decrease in the number and length of hyphae under certain conditions, as well as a weakening of the functions of actin at the hyphal tip. Furthermore, the absence of Spf1 also leads to abnormal vacuole morphology under hyphal induction conditions, which may also result in defects in hyphal development (Yu et al. 2019). All these observations indicate the important physiological role of Spf1 in the growth of *C. albicans*.

The endoplasmic reticulum-plasma membrane (ER-PM) contacts is one of the important cellular structures. Some endoplasmic reticulum is directly contacted with the plasma membrane through anchoring functions of tethering proteins such as Ist2, Tcb1, and Tcb3. This contact mainly functions in material exchange and information communication within the cell (Schulz and Creutz 2004; Chang et al. 2017; Wong et al. 2021). More specific studies have found that the function of this ER-PM contact covers many aspects, such as ion and lipid transport, signal transduction, endoplasmic reticulum reshaping, membrane transport, and regulation of hyphal formation. These functions are indispensable in the normal growth and host infection processes of *C. albicans* (Li et al. 2021; Yang et al. 2022).

It is known that the endoplasmic reticulum is an important storage site for calcium ions in cells, and research has shown that the absorption of calcium ions by the plasma membrane and the regulation of calcium ions by the endoplasmic reticulum is not only related to the direct transport channel calcium pump, but also to the ER-PM contact (Stefan et al. 2013). For example, in muscle cells, the contact between the endoplasmic reticulum and plasma membrane controls the release of calcium ions by interacting with voltage-gated calcium channels and ryanodine receptors, which is a key process for the excitation and contraction of muscle cells (Dixon and Trimmer 2023). This indicates the important and stable influence of ER-PM contacts on calcium ions. Recently, we found that after Spf1 loss, the infectivity of *C. albicans* to the host significantly decreased. However, the regulatory mechanism of Spf1 and its impact on the host’s immune response during this process are still unknown. Therefore, in this study, we investigated the role of Spf1 in the formation of ER-PM contacts, virulence factor secretion, and immune response. This study may build the connection between the calcium pump, ER-PM contacts and the immune response of *C. albicans* during its systemic infections.

## 2. Materials and methods

### 2.1. Fungal strains and plasmids

The fungal strains and plasmids used in this study were demonstrated in Table S1 and S2, respectively. All of the fungal strains were derived from the wild-type strain BWP17. The mutants were constructed by PCR-mediated homogenous recombination. The localisation strains were constructed by transformation of the corresponding localisation plasmids to the host strains.

### 2.2. Plasmid extraction

Plasmid-containing *E. coli* were streaked on LB solid medium and incubated at 30 °C for 12 h to obtain single colonies. A single colony was then picked and inoculated into a tube containing 3 mL of LB liquid medium, followed by overnight shaking at 37 °C. Subsequently, 1 mL of the culture was transferred to a 1.5 mL centrifuge tube, centrifuged at 12,000 r/min for 1 min, and the supernatant was discarded to collect the bacterial cells.

To the centrifuge tube, 200 μL of Solution I (1.8 g glucose, 0.6 g Tris-base, and 0.6 g EDTA dissolved in 200 mL distilled water) and 3 μL RNaseA were added. The tube was vortexed for 30 s (level 7) to fully suspend the cells and then incubated on ice for 6 min. Next, 200 μL of Solution II (88 mL distilled water, 2 mL 10 mol/L NaOH, and 10 mL 10% SDS) was added, and the tube was inverted 2–3 times and incubated on ice for 6 min. Following this, 200 μL of Solution III (29.4 g potassium acetate dissolved in 88 mL distilled water with 11.5 mL glacial acetic acid) was gently inverted 2–3 times and incubated on ice for 6 min.

The tube was then centrifuged at 4 °C at 12,000 r/min for 10 min, and 500 μL of the supernatant was transferred to a new 1.5 mL centrifuge tube. An equal volume of chloroform-isoamyl alcohol (24:1) was added, vortexed for 1 min (level 9), and centrifuged at 4 °C at 12,000 r/min for 10 min. Next, 400 μL of the supernatant was transferred to a new 1.5 mL centrifuge tube, and twice the volume of anhydrous ethanol (800 μL) was added to precipitate the DNA. The tube was then placed at −20 °C for 1 h. Following this, the tube was centrifuged at 4 °C at 12,000 r/min for 10 min, discard the supernatant and 1 mL of 75% ethanol was added to wash the DNA. Finally, the tube was centrifuged at 4 °C at 12,000 r/min for 10 min, discard the supernatant and the DNA was air-dried at room temperature and stored at −20 °C.

### 2.3. Gene transformation

#### 2.3.1. Lithium acetate transformation

Adjust the OD value of the fungal solution to 0.1 and incubate in 50 mL YPD liquid medium at 160 r/min and 30 °C for 4 h on a shaker. Subsequently, dissolve the plasmid in 40 μL sterile water and digest with the enzyme system for 1–2 h. (The enzyme system consists of 32 μL plasmid, 2 μL *BgrII*, 2 μL *PstI*, and 4 μL 10× H). Centrifuge the fungal solution at 4,200 r/min for 2 min, discard the supernatant, then add 5 mL TELiAc (4 mL H_2_O + 500 μL TE + 500 μL LiAc), centrifuge at 4,200 r/min for 2 min, discard the supernatant, and repeat twice. Resuspend the pellet in 300–400 μL TELiAc and aliquot into 1.5 mL EP tubes (100 μL fungal suspension per tube).

Add 10 μL salmon sperm DNA (SAS) to each EP tube (previously boiled for 8 min and placed on ice), add 10–20 μL enzyme-digested plasmid, and incubate at 30 °C and 60 r/min for 30 min. After incubation, add 700 μL PLATE to each EP tube (100 μL TE + 100 μL LiAc + 800 μL PEG), heat shock at 42 °C for 1 h, then centrifuge at 5,000 r/min for 2 min, discard the supernatant, add 1 mL sterile water for washing, repeat three times, and finally resuspend in 300 μL sterile water. Plate 100 μL on SC-His plates, incubate at 30 °C until single colonies appear, and perform transformation validation (Walther and Wendland 2003).

#### 2.3.2. Nucleic acid gel electrophoresis validation

Pick a single colony grown on selective medium into 3 mL SC-His liquid medium and incubate overnight for 12 h. Centrifuge 1 mL of the fungal culture at 12,000 r/min for 3 min and discard the supernatant. Add approximately 250 μL glass beads, 500 μL TENTS buffer, 250 μL chloroform-isoamyl alcohol solution (chloroform:isoamyl alcohol = 24:1), and 250 μL Tris-saturated phenol. Shake the mixture in a vortex oscillator (level 9) for 10 min to disrupt the fungal cells. Then, centrifuge at 12,000 r/min at 4 °C for 10 min, transfer 400 μL of the supernatant to a new 1.5 mL centrifuge tube, add 1 mL anhydrous ethanol, mix well, and incubate at −20 °C for 40 min for ethanol precipitation. Centrifuge under the same conditions, discard the supernatant, add 250 μL RNA digestion enzyme, incubate for 20 min, then add 70 μL 3 mol/L sodium acetate and 500 μL anhydrous ethanol, and incubate at −20 °C for 30 min for ethanol precipitation. Centrifuge under the same conditions, discard the supernatant, add 500 μL 75% ethanol for washing, centrifuge again, and resuspend the precipitate in 30–40 μL deionised water to obtain the genomic DNA.

Prepare the PCR system: forward primer (10 μmol/L) 1 μL, reverse primer (10 μmol/L) 1 μL, Template DNA 2 μL, PCR Mix (2×) 12.5 μL, ddH_2_O 8.5 μL. After PCR, agarose gel electrophoresis is performed to observe if the genomic DNA shows the same bands as the plasmid.

#### 2.3.3. Fluorescence validation

Pick a single colony grown on selective medium into 3 mL SC-His liquid medium and incubate overnight for 12 h. Take a small amount of the fungal culture to observe under a fluorescence microscope to check for the presence of red fluorescence.

### 2.4. Flow cytometry

#### 2.4.1. Preparation of single-cell suspension

Take WT, *spf1-/-*, and *SPF1C*-infected mouse kidneys after three days. Wash the kidney with PBS and use scissors to cut the tissues into 2–3 pieces. Transfer the tissue to HBSS medium containing 2 mmol/L EDTA and shake at 37 °C for 8 min. Subsequently, wash and chop the tissue pieces into smaller fragments, transfer them to a digestion medium, and culture at 37 °C with shaking at 150 r/min for 40 min. Wash the digested tissue with PBS and filter through a 200-mesh sieve. Centrifuge at 1,700 r/min for 3 min and wash with PBS again. Resuspend the pellet in 1 mL PBS to obtain a single-cell suspension (Li et al. 2022).

#### 2.4.2. Perform immunofluorescence staining and flow cytometry analysis

Control group: Mouse kidney single-cell suspension without fungal, no antibodies; Mouse kidney single-cell suspension without fungal, with PE-CD45, FITC-Ly6G, APC-CD11b antibodies; Mouse kidney single-cell suspension without fungal, with PE-CD45 antibody; Mouse kidney single-cell suspension without fungal, with APC-CD11b antibody; Mouse kidney single-cell suspension without fungal, with FITC-Ly6G antibody. Experimental group: Add PE-CD45, FITC-Ly6G, and APC-CD11b antibodies to the mouse kidney samples infected with different genotypes of *C. albicans* (Gullotta et al. 2023).

#### 2.4.3. Data analysis

Analyse the data based on the fluorescence channels corresponding to PE (Yel-B), FITC (Green-B), and APC (Red-R) using FlowJo (Version 10.8.1, BD Biosciences, Ashland, OR, USA), and export the analysed data and images.

### 2.5. Kidney frozen section and immunofluorescence staining

#### 2.5.1. Fixation and dehydration of kidney tissue

Take fresh kidney and place it in 4% paraformaldehyde (PFA) at 4 °C for 12 h. Transfer the kidney to a 15% sucrose solution and continue incubating at 4 °C for 12 h. Subsequently, transfer the kidney to a 30% sucrose solution and continue incubating at 4 °C for 12 h. Finally, transfer the kidney to a mixture of 30% sucrose and OCT (1:1) and continue incubating at 4 °C for 12 h. After fixation and dehydration, place it in an embedding box. Use OCT to freeze the kidney at −20 °C for 20 min. Subsequently, section the frozen kidney into 8 μm slices using a cryostat and store at −20 °C (Foley and Bard 2002).

#### 2.5.2. Immunofluorescence staining

Remove the mouse kidney sections from the −20 °C freezer and bake them on a slide warmer at 60 °C for 1 h. Place the sections in a wash box containing 1× PBS (ensure the liquid covers the sections), and immerse them 3 times for 5 min each to dissolve the embedding agent OCT. Next, add 4% PFA to the tissue sections, ensuring they are completely covered, and fix for 30 min. Then, add a sufficient volume of PBST (1× PBS + 0.1%–0.3% TritonX-100) and incubate at room temperature for 15 min. After fixation, use a hydrophobic pen to draw a circle around the kidney sections. Within the hydrophobic circle, add a sufficient amount of 5% bovine serum albumin (BSA) diluted in PBST, and incubate at room temperature for 1 h.

After incubation, wash the sections once with PBST for 5 min, discard the blocking solution. Then, transfer the sections to a wet box containing an appropriate amount of deionised water, add TNF-α dye within the hydrophobic circle, and incubate in the dark at room temperature for 30 min. After incubation, wash the sections 3–5 times with 1× PBS for 10 min each, then add DAPI and incubate at room temperature for 3–5 min, followed by a 5-min wash with 1× PBS. After staining, add 50–80 μL of anti-fade mounting medium, place a cover slip over the sections, and seal the edges with nail polish, then examine the sections using a confocal microscope.

### 2.6. Quantitative real-time PCR

#### 2.6.1. RNA extraction

The mouse kidney tissues were transferred to 1.5 mL tubes without ribonuclease. After that, 1 mL of Trizol was added and mixed thoroughly. The cells were then lysed using a tissue homogeniser and left to stand at room temperature for 5 min to allow complete separation of the nucleic acid-protein complexes. Subsequently, 0.2 mL of chloroform was added, followed by vigorous shaking for 30 s, and then the mixture was left to stand for 2–3 min before centrifugation at 12,000 r/min for 15 min at 4 °C. After centrifugation, the sample was separated into three layers, with RNA mainly concentrated in the upper aqueous phase. Carefully aspirate the supernatant into a new ribonuclease-free tube and add 0.5 mL of isopropanol. Gently mix the liquid in the tube and let it stand at room temperature for 10 min. The tube was then centrifuged at 12,000 r/min for 10 min at 4 °C. After discarding the supernatant, 1 mL of 75% ethanol (diluted with ribonuclease-free water) was added to the precipitate and shaken to fully wash the precipitate. After centrifugation at 12,000 r/min for 5 min at 4 °C, the supernatant was discarded, and the precipitate was air-dried. Finally, the extracted RNA was stored in a −80 °C freezer or used directly for reverse transcription. To confirm the success of RNA extraction, 2 μL of the extracted RNA was mixed with 1 μL of loading buffer and analysed using agarose gel electrophoresis. The electrophoresis results were observed, and the presence of clear bands indicated successful RNA extraction.

#### 2.6.2. Inverse transcription

The RNA was dissolved in ribonuclease-free water at 65 °C for 10–15 min. A reverse transcription RNA mixture was prepared by adding 40 μL of the RNA extract to 1.5 μL of oligo dT and 1.5 μL of dNTP mixture. The mixture was incubated at 65 °C for 5 min, followed by a 2-min incubation on ice. The RNA mixture was then mixed with 2 μL of RNase inhibitor (RPI), 3 μL of MLV-Reverse Transcriptase, and 12 μL of MLV 5× buffer for reverse transcription PCR. The resulting cDNA was stored at −80 °C.

#### 2.6.3. Quantitative real-time PCR system

Prepare the qPCR reaction system as follows: 2 μL of forward primer, 2 μL of reverse primer, 2 μL of cDNA, 10.5 μL of mix, and 3.5 μL of ribonuclease-free water. Place the prepared system into the qPCR instrument for analysis. The primers used are for Actin, IL-1β, IL-4, IL-6, IL-10, IFN-γ, and TGF-β1, designed to detect different inflammatory factors. After obtaining the results, perform relative quantification using the 2^−ΔΔCt^ method to calculate their relative expression levels (Winer et al. 1999).

### 2.7. Electron microscopy observation

Under the electron microscope, the ER-PM contact of *C. albicans* was observed. Subsequently, Adobe Photoshop was used to label the contacted endoplasmic reticulum with the plasma membrane, the endoplasmic reticulum contacted with the nuclear membrane, and the free endoplasmic reticulum in the cytoplasm with different colours (Stefan et al. 2013). Following this, Image J (Version 1.54f National Institutes of Health, Bethesda, MD, USA) was employed to measure the relative lengths of the endoplasmic reticulum contacted with the plasma membrane and the free endoplasmic reticulum in the cytoplasm. After analysing the ratio of lengths, create a bar graph using GraphPad Prism (Version 10.2.0, GraphPaD, San Diego, CA, USA) and perform statistical analysis of the experimental data using SPSS to determine significant differences.

### 2.8. The secretion capacities of protease and phospholipase

#### 2.8.1. *The ability of* C. albicans *to secrete phospholipase*

Adjust the OD value of the fungal culture to 0.5 using a spectrophotometer. Then, spot 1.5 μL of the culture onto solid yolk medium. Incubate at 37 °C for 3 d until distinct hydrolysis zones appear. Photograph the plates and measure the diameters of the colonies and hydrolysis zones. Use Adobe Photoshop to mark the hydrolysis zones and analyse the ratio of the diameter of the hydrolysis zone to the diameter of the colony. Create a bar graph using GraphPad Prism and perform statistical analysis of the experimental data using SPSS to determine significant differences (Price et al. 1982; Nciki et al. 2020).

#### 2.8.2. *The ability of* C. albicans *to secrete protease*

Adjust the OD value of the fungal culture to 0.5 using a spectrophotometer. Inoculate the adjusted culture into 20 mL of BSA liquid medium. Incubate at 37 °C on a shaker. After 12 h of incubation, take 1 mL of the culture from each flask, centrifuge at 12,000 r/min for 3 min, collect the supernatant, and store it at −20 °C (Hube et al. 1997). Perform SDS-PAGE gel electrophoresis on the supernatant, adding 5 μL of sample to each well, and stain with Coomassie brilliant blue. Photograph and analyse the protein bands. Use Image J for greyscale analysis. Create a bar graph using GraphPad Prism and perform statistical analysis of the experimental data using SPSS to determine significant differences.

### 2.9. Assays of candidalysin secretion

#### 2.9.1. Acquisition and detection of antibodies

Mix 500 μL of candidalysin antigen, 50 μL of BCG Vaccine, and 450 μL of Freund’s adjuvant. Emulsify the mixture by repeatedly drawing it in and out of a syringe until it appears milky white. Inject 200 μL of the mixture subcutaneously into each mouse once a week for three weeks. After three weeks, blood from the mice was collected via eye bleeds and the blood was centrifuged to obtain antibodies. Use the candidalysin antigen for coating, and HRP Goat Anti-Mouse as the secondary antibody to determine antibody titres. Select the antibody with the highest titre for further experiments.

#### 2.9.2. Determination of antigen content

Cultivate *C. albicans* overnight in YPD liquid medium. Adjust the OD value to 0.1 before adding it to 50 mL of N-acetylglucosamine medium with 50 μL of Uri. Incubate at 37 °C for 18 h, then centrifuge at 12,000 r/min for 3 min to collect the supernatant for BCA assay. Adjust the protein concentration using a standard curve and coat the supernatant onto a 96-well plate, incubating at 4 °C for 12 h. ELISA was used to measure candidalysin content among different genotypes, detecting fluorescence at OD = 450 nm with an ELISA reader. Create a bar graph using GraphPad Prism and perform statistical analysis of the experimental data using SPSS to determine significant differences.

### 2.10. C. albicans *infection* in vitro

#### 2.10.1. *Construction and verification of* in vitro *infection model*

To observe the effects of different *C. albicans* genotypes on systemic infection in epithelial cells, we used bEnd.3 mouse brain endothelial cells to simulate the physiological barrier defence against pathogen invasion (Ferro et al. 2020).

Transwell filters (8 μm pore size) were placed in a 24-well plate. Since the growth area of each well is 0.33 cm^2^, the initial cell concentration was adjusted to 2 × 10^4^ cells/200 μL (Rabanel et al. 2020). 200 μL of cell suspension was added to the upper chamber of the Transwell, and 1 mL of complete DMEM medium (DMEM + 1% Penicillin-Streptomycin Solution and 10% Serum) was added to the lower chamber. The cells were cultured, and the medium in both chambers was changed every two days for the first seven days, and then every day until the tenth day to complete the model construction.

After the tenth day, the model construction was completed, and the formation of tight junctions between endothelial cells was observed under an optical microscope to determine the barrier integrity. Subsequently, the fluorescence permeability of the model was determined by replacing the medium in both chambers with serum-free DMEM and adding 5 μL of 2 mg/mL fluorescein isothiocyanate (FITC) to the upper chamber. Complete fluorescence and non-fluorescence control experiments were conducted (Wegener and Seebach 2014). After incubating at 37 °C in the dark for one hour, the lower chamber medium from the experimental and control groups was divided into three portions for fluorescence intensity measurement to assess the integrity of the barrier.

#### 2.10.2. C. albicans *infection*

After confirming the barrier integrity of the model, infect the bEnd.3 mouse brain endothelial cell models with different genotypes of *C. albicans*. Adjust the OD value of the fungal suspension to 0.2 before co-culture. After one day of co-culture, stain the models with calcofluor white (CFW, 10 mg/L, Sigma, USA) and Alexa Fluor 594-labelled concanavalin A (5 mg/L, Thermo Fisher, USA). Finally, use confocal microscopy to observe the models.

#### 2.10.3. Data analysis

Use Image J to analyse the fluorescence intensity of red and blue fluorescence. Use GraphPad Prism to create a bar graph using the ratio of red to blue fluorescence intensity as an indicator of the tight junction integrity of the *in vitro* model. Perform statistical analysis of the experimental data using the SPSS software to determine significant differences.

### 2.11. C. albicans *infection* in vivo

#### 2.11.1. *Transformation of* URA3 *gene*

Use the lithium acetate transformation method to transform the pLUBP plasmid into *C. albicans* to restore the ability of infection. Single colonies grown on SC-Uri selection medium are picked to extract the genome. PCR is performed on the inserted genes, and agarose gel electrophoresis is used to verify the successful transfer of the *URA3* gene using the plasmid as a control (Brand et al. 2004; Liang et al. 2018).

#### 2.11.2. C. albicans *infection*

Adjust the OD value of the fungal culture to 0.5 using physiological saline. Inject the adjusted culture into mice via tail vein injection. Record the time and number of mouse deaths. Periodically extract the kidneys for grinding, dilute the tissue, and use the spread plate method to determine *C. albicans* content. Fix the kidneys, prepare tissue sections using paraffin embedding, and perform Haematoxylin and Eosin (HE) staining to observe the distribution of inflammation.

#### 2.11.3. Data analysis

Use SPSS to plot mouse survival curves. Use GraphPad Prism to create a bar graph of *C. albicans* content in mouse kidneys. Perform statistical analysis of the experimental data using SPSS to determine significant differences.

## 3. Results

### 3.1. *Deletion of* SPF1 *attenuates kidney inflammation during systemic infection*

**Figure 1.**
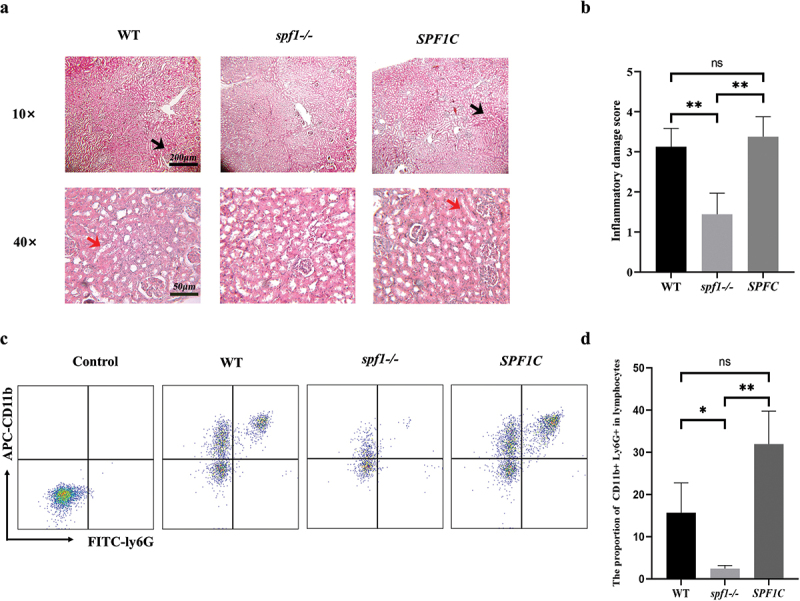
Influence of *SPF1* deletion on inflammatory response. (a) Three days after tail vein injection, mouse kidneys were paraffin-sectioned and stained with HE (black arrows indicate inflammatory sites under a 10× microscope and red arrows indicate hyphae under a 40× microscope). (b) Scoring criteria for inflammation: no damage to renal tubules (score of 0), <25% damage to renal tubules (score of 1), 25%–50% damage to renal tubules (score of 2), 50%–75% damage to renal tubules (score of 3), 75%–100% damage to renal tubules (score of 4) (*n* = 3, ns: *p* > 0.05, *: *p* < 0.05, **: *p* < 0.01). (c) Under the conditions of SSC/FSC, SSC/PE-CD45, and APC-CD11b/FITC-ly6G, single cells, lymphocytes, and neutrophils were successively gated to obtain the proportion of neutrophils in kidney single-cell suspensions after infection with different genotypes of strains. (d) Proportion of CD11b + Ly6G + cells in lymphocytes (*n* = 3, ns: *p* > 0.05, *: *p* < 0.05, **: *p* < 0.01).

During *C. albicans* infection, hyphae invade and damage multiple organs, with the kidneys being the most severely affected (Jawale and Biswas 2021). To investigate the impact of *SPF1* gene deletion on inflammatory response, we used 5-week-old female ICR mice, injected different genotypes of *C. albicans* into their tail veins, and then sampled the kidneys on the third day for paraffin sectioning and HE staining. Examination of kidney sections revealed severe inflammation in the renal tissue after infection with the wild-type strain or the *SPF1*-complemented strain (*SPF1C*). However, there was almost no apparent inflammatory response after *spf1-/-* infection (Figure 1a). Observation under a 40× magnification revealed that while hyphal colonisation was present in renal tissue after infection, the growth of hyphae was significantly inhibited after *SPF1* gene deletion (Figure 1a). Subsequently, we assessed the specific renal inflammation by calculating the percentage of damaged glomeruli (including glomerular inflammation, cell proliferation, and tubular deformation) in each photograph relative to the total number of glomeruli (Figure 1b). The results showed that mice infected with WT and *SPF1C* exhibited significant inflammation in the kidneys, including extensive glomerular cell proliferation, a significant presence of red blood cells in the interstitial tissue, and severe inflammatory cell infiltration and cell swelling. In contrast, mice infected with *spf1-/-* had milder renal symptoms, with most glomeruli remaining normal.

Subsequently, to explore the number of immune cells at the site of kidney inflammation, we used flow cytometry and selected three antibodies labelled with PE-CD45, FITC-Ly6G, and APC-CD11b to screen for neutrophils from kidney tissue (Hasenberg et al. 2015). CD45 is a tyrosine phosphatase widely expressed on the surface of all white blood cells, including neutrophils. Simultaneous staining with the neutrophil marker Ly6G and the macrophage marker CD11b can effectively separate neutrophils from lymphocytes (Charbonneau et al. 1988; Daley et al. 2008; Pillay et al. 2013). We selected the kidneys of mice three days after tail vein injection of the fungal suspension, prepared single-cell suspensions, and stained them for flow cytometry analysis, with the results as follows (Figure 1c,d). Through flow cytometry results and data analysis, we found that in the results of infection with the wild-type strain, the proportion of neutrophils was higher. Conversely, in the results of infection with the *spf1-/-* genotype, there was a significant decrease in the proportion of neutrophils to lymphocytes. Similarly, the proportion of neutrophils returned to a level similar to that of the wild type after the complementation of the *SPF1* gene in *C. albicans*. Overall, the inflammatory response in the kidneys of mice infected with *C. albicans* is greatly reduced after *SPF1* gene deletion.

### 3.2. *Deletion of* SPF1 *reduces the production of inflammatory cytokines*

**Figure 2.**
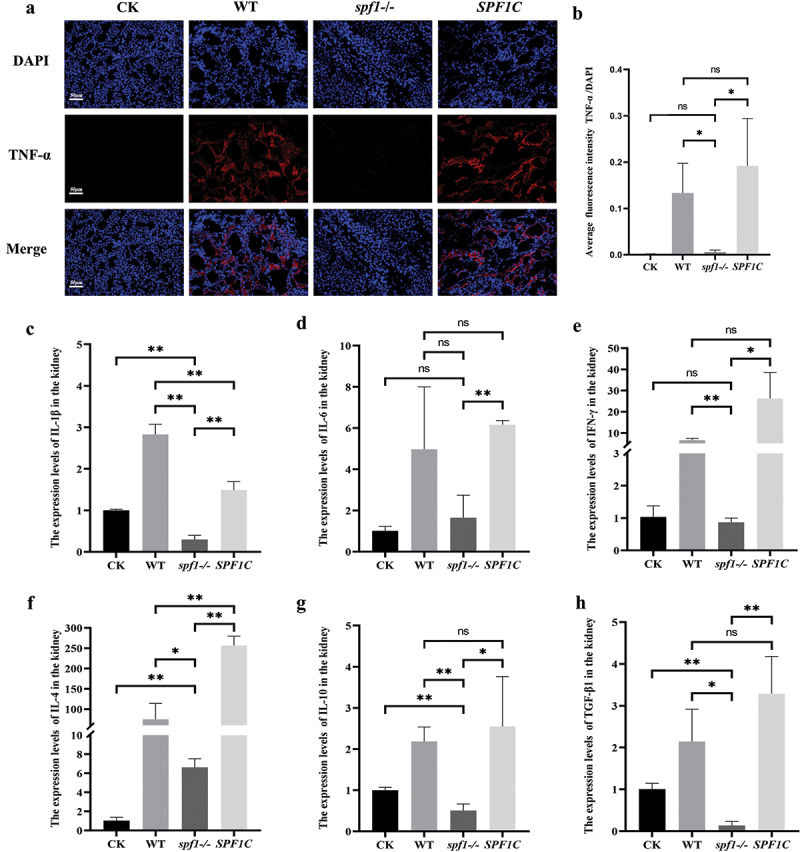
Influence of *SPF1* deletion on expression of inflammatory cytokines. (a) Confocal microscopy images of kidney sections: the nuclear is labeled with blue fluorescence, and the cytokine tnf-α is labeled with red fluorescence. (b) Average fluorescence intensity of TNF-α/DAPI (*n* = 3, ns: *p* > 0.05, *: *p* < 0.05, **: *p* < 0.01). (c–h) Expression levels of different inflammatory factors in the kidneys, including IL-1β, IL-6, IFN-γ, IL-4, IL-10, TGF-β1 (*n* = 3, ns: *p* > 0.05, *: *p* < 0.05, **: *p* < 0.01).

TNF-α is a pro-inflammatory cytokine that can activate phagocytic cells (such as macrophages and neutrophils) during fungal infections, promoting inflammation at the infection site and stimulating the production of other cytokines and chemokines, thereby enhancing the anti-fungal immune response (Herring et al. 2005). To further investigate the effect of *SPF1* deletion in cytokine-level immune responses, we conducted frozen sectioning and immunofluorescence staining of mouse kidneys infected with *C. albicans* after three days. As revealed by fluorescence microscopy and intensity analysis, the mice infected with the wild-type or the *SPF1C* strain showed significant distribution of TNF-α signal, while the mice infected with the *spf1-/-* strain exhibited weaker TNF-α distribution, with the red fluorescence almost disappearing (Figure 2a,b).

To further confirm the difference in expression levels of other cytokines (such as pro-inflammatory factors IL-1β, IL-6, IFN-γ, anti-inflammatory factors IL-4, IL-10, TGF-β1) in the kidneys during infection with different genotypes of *C. albicans*, we conducted real-time fluorescence quantitative PCR (qPCR) experiments (Nailis et al. 2006). Subsequently, the relative expression levels were calculated using the 2^−ΔΔCt^ method (Figure 2c–h, Figure S1). As compared to the blank control group (CK) and the *spf1-/-* group, the levels of most cytokines significantly increased in the kidneys of mice infected with the WT and *SPF1C* strains. In addition, the trends of these cytokines were not completely consistent between WT and *SPF1C*, which may be due to the different expression levels of *SPF1* in the two strains. Nevertheless, the levels of cytokines in mice infected with *spf1-/-* are significantly lower than that in the mice infected with the wild-type strain.

### 3.3. *Deletion of* SPF1 *strongly impairs formation of ER-PM contacts*

**Figure 3.**
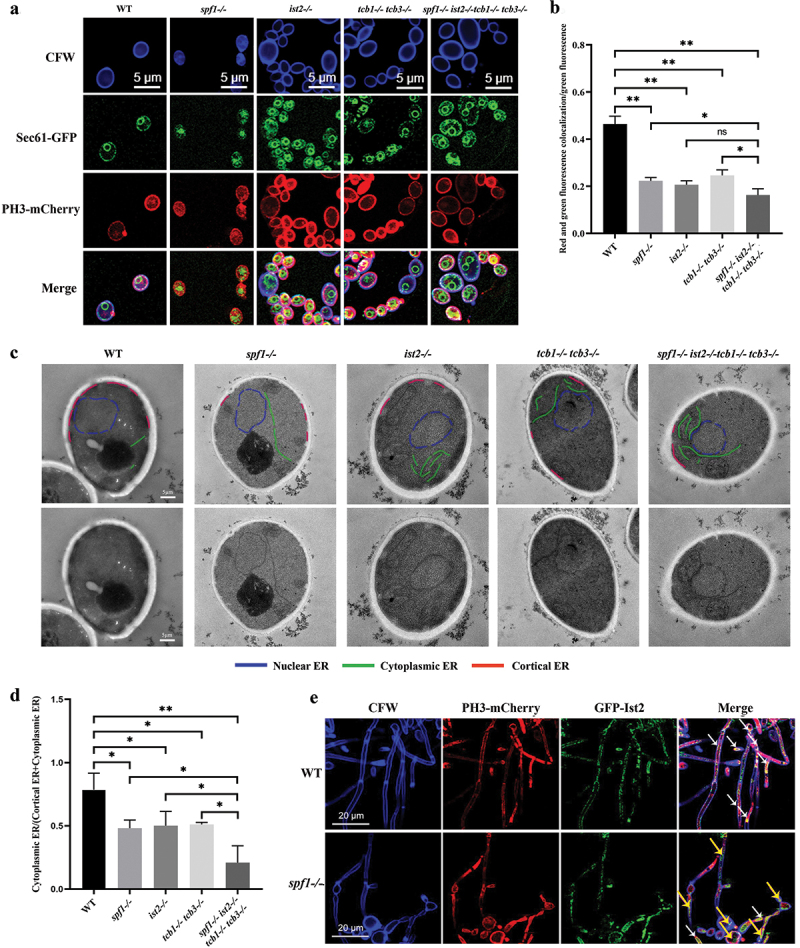
Influence of Spf1 and ER-PM tethering protein loss on ER-PM contact. (a) Confocal microscopy image of *Candida albicans*: the cell wall is labeled with blue fluorescence, the plasma membrane is labeled with red fluorescence, and the endoplasmic reticulum localized protein Sec61 is labeled with green fluorescence. (b) Ratio of co-localization area of red and green fluorescence to the area of green fluorescence alone in different genotypes (*n* = 3, ns: *p* > 0.05, *: *p* < 0.05, **: *p* < 0.01). (c) Distribution of endoplasmic reticulum in *C. albicans*: the blue line represents the nuclear endoplasmic reticulum, the green line represents the cytoplasmic endoplasmic reticulum, and the red line represents the endoplasmic reticulum contacted to the plasma membrane. (d) Ratio of the cytoplasmic endoplasmic reticulum to (endoplasmic reticulum contacted to the plasma membrane + cytoplasmic endoplasmic reticulum) in different genotypes (*n* = 3, ns: *p* > 0.05, *: *p* < 0.05, **: *p* < 0.01). (e) Confocal microscopy image of *C. albicans* hyphae: The cell wall is labeled with blue fluorescence, the plasma membrane is labeled with red fluorescence, and the ER-PM tethering protein Ist2 is labeled with green fluorescence (white arrows indicate overlap of red and green fluorescence, purple arrows indicate green fluorescence alone). The fungal hyphae were induced in RPMI-1640 medium at 37 °C for 4 h, followed by confocal microscopy.

To investigate the relationship between Spf1 and ER-PM tethering proteins (such as Ist2 and Tcb1/3), we constructed the ER-PM contact mutants *ist2-/-*, *tcb1-/-tcb3-/-*, and s*pf1-/-ist2-/-tcb1-/-tcb3-/-*. Initially, we used the channel Sec61 involved in protein transport and translocation on the endoplasmic reticulum as an endoplasmic reticulum marker, and the PH3 membrane-localised protein as a plasma membrane marker (Fonzi 2009). Following CFW staining of the strains, the experimental results were obtained by calculating the ratio of the area co-located with red fluorescence and green fluorescence to the area where green fluorescence was present alone (Figure 3a,b). The results showed a significant decrease in the area of co-localisation of the endoplasmic reticulum and plasma membrane in *C. albicans* after the loss of the *SPF1* gene, and the level of this loss was similar to that after the loss of Ist2 and Tcb1/3 proteins. We then used electron microscopy to observe the internal structure of these *C. albicans* (Figure 3c). In the figure, blue labelling represents the endoplasmic reticulum in contact with the nuclear membrane, green labelling represents the free endoplasmic reticulum in the cytoplasm, and red labelling represents the endoplasmic reticulum in contact with the plasma membrane. It can be seen that the gene loss leads to the loss of ER-PM tethering proteins, resulting in the aberrant localisation of the endoplasmic reticulum and greatly altering the morphology of the peripheral endoplasmic reticulum network, leading to the accumulation of free endoplasmic reticulum (Stefan et al. 2013). The results of comparing the ratio of the lengths of the endoplasmic reticulum in the cytoplasm and the endoplasmic reticulum contacted to the plasma membrane in different images are shown in Figure 3d. The impact of Spf1 loss on ER-PM contact is comparable to the direct loss of tethering proteins, and these effects can be superimposed in the *spf1-/-ist2-/-tcb1-/-tcb3-/-* strain, leading to a more pronounced decrease in ER-PM contacts.

To further investigate the relationship between the *SPF1* gene and the ER-PM tethering protein, we used the ER-PM tethering protein Ist2 as a marker for confocal microscopy (Figure 3e) (Wolf et al. 2012). The results showed that in the hyphal cells of the WT genotype, there was a considerable overlap between green and red fluorescence (indicated by white arrows), indicating that in the wild-type strain, the ER-PM tethering protein Ist2 mostly co-localises with the plasma membrane. However, in the *SPF1* gene-deficient strain, there are many individual green fluorescence (indicated by purple arrows, Figure 3e). Similar results were observed in the yeast cells. While the WT cells had most of GFP-Ist2 co-localised with the PH3-mCherry (indicating the PM), the *spf1-/-* cells had abundant GFP-Ist2 in the cytosol rather than at the PM (indicated by the purple arrows, Figure S2). Hence, the Ist2 protein has dissociated from its original plasma membrane contact site, losing its contact function in *spf1-/-*. These results suggest that loss of Spf1 leads to abnormalities in tethering proteins such as Ist2, resulting in a decrease in ER-PM contact and functional loss.

### 3.4. Loss of Spf1 and related ER-PM tethering proteins attenuates secretion of virulence factors

*Candida albicans* infects host cells by adhering to epithelial cells and secreting hydrolytic enzymes, candidalysin, and other virulence factors (Fan et al. 2013). Following phase transition and adhesion, *C. albicans* can penetrate host cells more deeply by SAP and phospholipase to disrupt the host cell membrane for infection. Aspartyl protease can cleave covalently linked cell wall proteins, while phospholipase can directly degrade the phospholipid bilayer of the host cell membrane (Niewerth and Korting 2001; Schild et al. 2011). Additionally, the precursor protein (Ece1p) encoded by the *C. albicans ECE1* gene undergoes specific protein hydrolysis by Kex2p, followed by further processing with the carboxypeptidase Kex1p to produce mature candidalysin. This toxin can also disrupt plasma membrane integrity and activate the immune response (Moyes et al. 2016).

**Figure 4.**
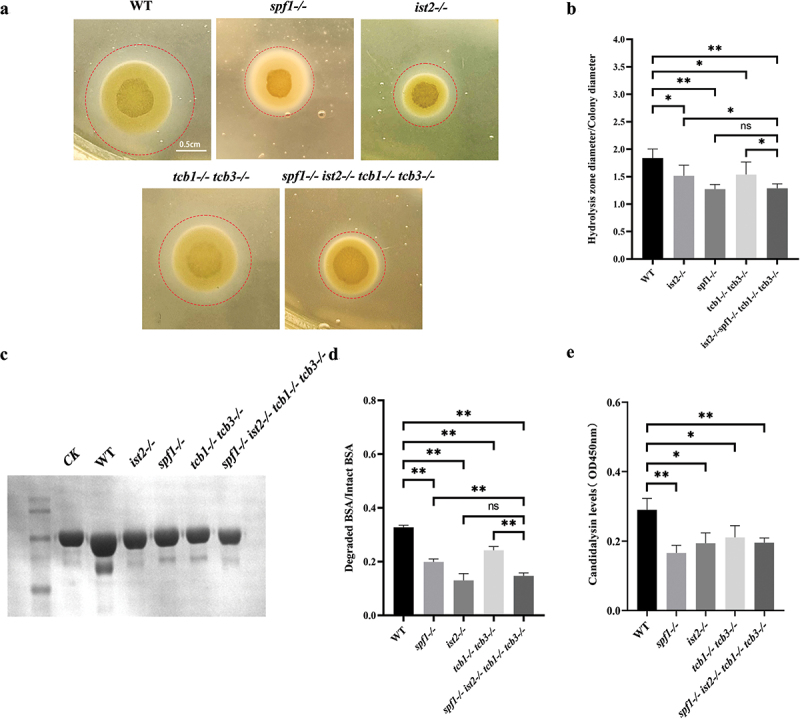
Influence of Spf1 and ER-PM tethering protein loss on secretion of the fungal virulence factors. (a) Results of cultivation of different strains on egg yolk medium (red dashed line represents hydrolysis zone). (b) Ratio of hydrolysis zone diameter to colony diameter of different strains (*n* = 3, ns: *p* > 0.05, *: *p* < 0.05, **: *p* < 0.01). (c) Protein electrophoresis image of different strains cultured in BSA medium for 12 h. (d) Ratio of degraded BSA protein to intact BSA protein in different strains (*n* = 3, ns: *p* > 0.05, *: *p* < 0.05, **:*p* < 0.01). (e) ELISA experiment shows the fluorescence intensity of candidalysin (OD 450 nm, *n* = 3, ns: *p* > 0.05, *: *p* < 0.05, **: *p* < 0.01).

To investigate the changes in virulence factors of *C. albicans* after the decrease in ER-PM contact levels, we cultured the strains on egg yolk solid medium and analysed the phospholipase levels of different genotypes. The results of egg yolk solid medium hydrolysis circles showed that the colonies of the WT strain were the largest and had the most pronounced hydrolysis circles, while those of the *spf1-/-*, *ist2-/-*, and *spf1-/-ist2-/-tcb1-/-tcb3-/-* strains had smaller hydrolysis circles and colony diameters (Figure 4a). Statistical analysis further revealed that compared with WT, the *spf1-/-*, *ist2-/-*, *tcb1-/-tcb3-/-*, and *spf1-/-ist2-/-tcb1-/-tcb3-/-* strains had a significant decrease in phospholipase hydrolytic activity (Figure 4b). These results indicated that the defect in ER-PM contact has a significant impact on the level of phospholipase.

SAP degrades many host proteins, such as immunoglobulins and bovine serum albumin (BSA). To investigate the effect of ER-PM contacts on SAP secretion, we used BSA to detect the level of SAP (Costa et al. 2010). After co-culturing BSA with different strains for 12 h, the supernatants were collected for protein gel electrophoresis. The results showed that the mutants had decreased capacity to hydrolyse BSA as compared with the wild type. During *C. albicans* infection, the peptide toxin candidalysin, which is used for cell lysis, can also be secreted. Consistently, as revealed by enzyme-linked immunosorbent assay (ELISA), the four mutants also exhibited lower levels of secreted candidalysin than the WT strain (Figure 4e). Subsequently, we employed qPCR to specifically examine the expression levels of the virulence factors secreted aspartic proteases (SAP) and lipases in both WT and *spf1-/-* strains. Our findings revealed no significant difference in the expression levels of the tested four virulence factor-encoding genes (i.e. *SAP1*, *SAP2*, *LIP1*, and *LIP2*) between the two genotypes (Figure S3). These results indicated that deletion of the *SPF1* gene during infection led to a decrease in secretion of *C. albicans* virulence factors, and this reduction is not caused by a decrease in their expression levels. This suggested that loss of Spf1 caused a decrease in ER-PM contact by affecting the localisation of some ER-PM tethering proteins, leading to further attenuation of virulence factor secretion.

### 3.5. Loss of Spf1 and related ER-PM tethering proteins attenuates secretion of GPI-anchored proteins

The above experiments showed that the loss of Spf1 and ER-PM tethering proteins reduced the level of virulence factors in *C. albicans*. We then selected the important GPI-anchored protein Hwp1 in *C. albicans* to investigate the specific secretion process affected by the loss of Spf1 and tethering proteins. Hwp1 is an important adhesin in *C. albicans*, aiding in the adhesion of the fungus to host epithelial cells and playing a key role in hyphal development and infection dissemination (Maras et al. 2021; Arita et al. 2022). Since Hwp1 acts on the surface of the cell wall, it can also be used as an indicator for the transport of cell wall proteins to explore the effects and differences in secretion pathways (Yu et al. 2014).

**Figure 5.**
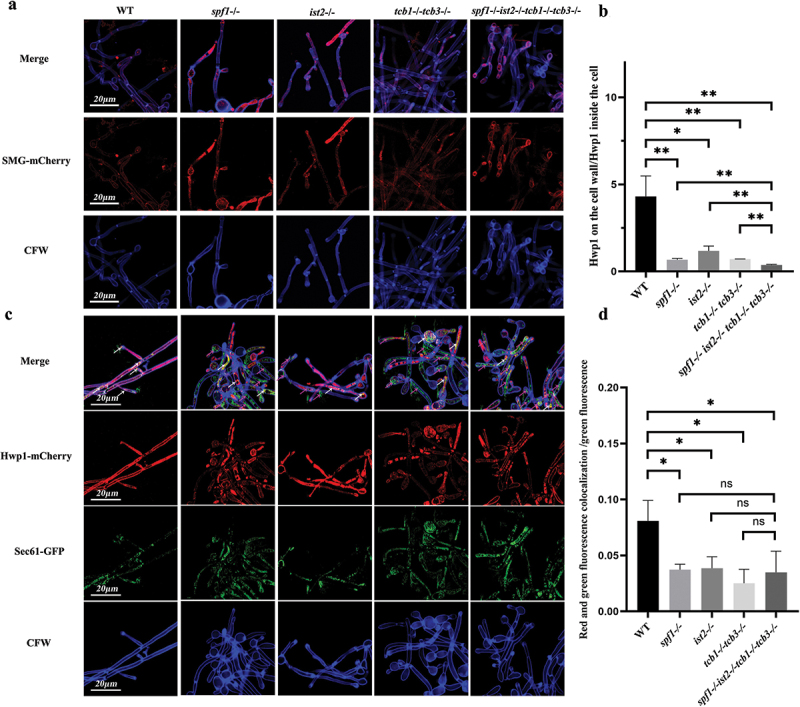
Influence of Spf1 and ER-PM tethering protein loss on cell wall localisation of gpi-anchored proteins. (a) Distribution of gpi-anchored proteins in hyphae of different genotypes: The cell wall is labeled with blue fluorescence, and the GPI-anchored proteins are labeled with red fluorescence. (b) Ratio of GPI fluorescence intensity on the cell wall to that inside the cell in hyphae of different genotypes (*n* = 3, ns: *p* > 0.05, *: *p* < 0.05, **: *p* < 0.01). (c) Colocalization of Hwp1 with the endoplasmic reticulum in hyphae of different genotypes: The cell wall is labeled with blue fluorescence, the Hwp1 is labeled with red fluorescence, and the endoplasmic reticulum localized protein Sec61 is labeled with green fluorescence (white arrows indicate overlap of red and green fluorescence, purple arrows indicate green fluorescence alone). (d) Ratio of the cell wall-localized Hwp1-mCherry to the intracellularly localized Hwp1-mCherry (*n* = 3, ns: *p* > 0.05, *: *p* < 0.05, **: *p* < 0.01).

To study the effect of ER-PM contact loss on the GPI anchoring of Hwp1, we constructed the plasmid SMG (signal peptide-mCherry-GPI anchoring sequence) for GPI labelling. This plasmid enables the GPI anchoring sequence of Hwp1 to be visualised with red fluorescence. Concurrently, Calcofluor White (CFW) was used to stain the *C. albicans* hyphae and cells (Figure 5a). The images show that in the WT, GPI is primarily distributed on the cell wall, overlapping with the blue fluorescence of CFW. However, in other deficient strains, a significant amount of red fluorescence was localised in the cytoplasm, suggesting that Hwp1 remains in the cytoplasm and is not successfully secreted externally to perform its anchoring function. After that, we used Image J to analyse the fluorescence intensity of the images and performed a significant analysis of the ratio of red fluorescence intensity on the cell wall to that in the cytoplasm. The results are shown in the Figure 5b. It was indicated that the cell wall-distributed GPI was significantly higher in the WT genotype than in other gene-deleted strains. Moreover, compared to single gene-deleted strains, the *spf1-/-ist2-/-tcb1-/-tcb3-/-* genotype exhibited a more pronounced decrease in the GPI secretion level, suggesting that the loss of ER-PM contacts strongly impaired secretion and cell wall anchoring of GPI.

To further confirm the impairment of Hwp1 secretion, we labelled Hwp1 with red fluorescence and the ER protein Sec61 with green fluorescence, and performed confocal microscopy of different mutants. As shown in Figure 5c, the Hwp1-mCherry fluorescence was mainly co-localised with the CFW fluorescence (indicated by white arrows) in the WT. In contrast, Hwp1-mCherry was abundantly co-localised with the Sec61-GFP fluorescence (indicated by purple arrows). Image J analysis further confirmed that the WT strain had much higher levels of cell wall-localised Hwp1 than the mutants (Figure 5d). These results suggested that after Spf1 and related ER-PM contacts were required for maintaining Hwp1 secretion and anchoring to the cell wall.

### 3.6. Spf1 and related ER-PM tethering proteins are required for successful invasion and systemic infection

**Figure 6.**
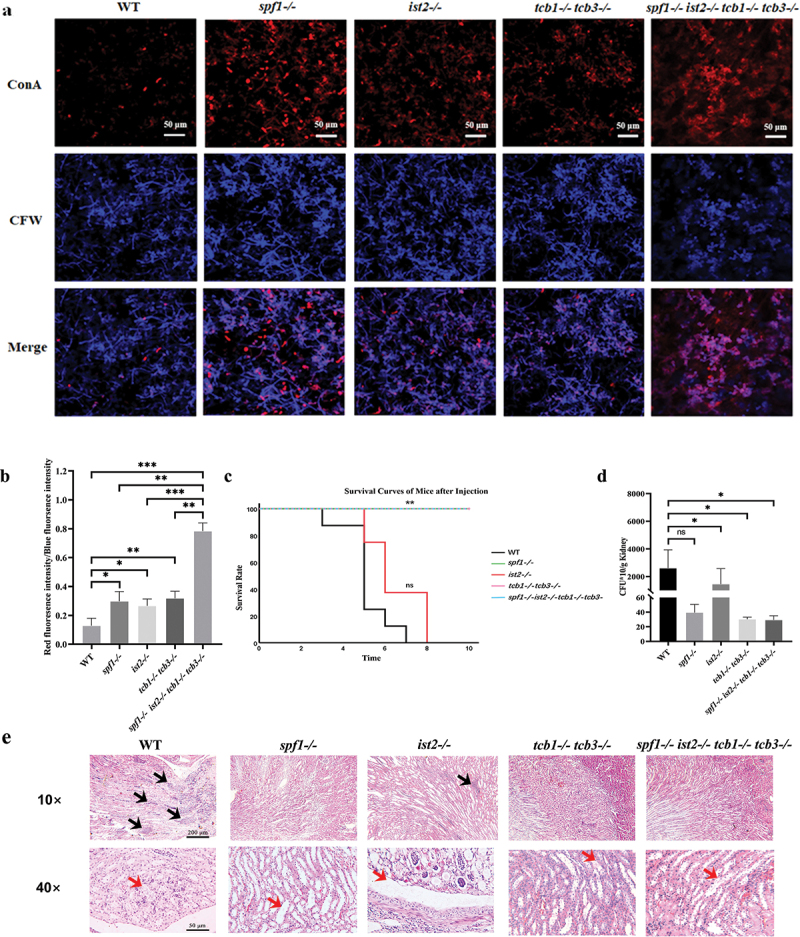
Influence of Spf1 and ER-PM tethering proteins loss on *Candida albicans* infection. (a) Endothelial layer disruption: the fungal cell wall is labeled with blue fluorescence, while the hyphae that failed to penetrate endothelial cells are stained by Alexa 594-labeled concanavalin a (ConA) with red fluorescence. (b) Ratio of red fluorescence intensity to blue fluorescence intensity after infection with different genotypes (*n* = 3, ns: *p* > 0.05, *: *p* < 0.05, **: *p* < 0.01). (c) Mouse survival curve after tail vein injection (*n* = 8, duration = 10 d). (d) Three days after tail vein injection, mouse kidney homogenates were spread on plates, and the number of single *C. albicans* colonies was counted (*n* = 3, ns: *p* > 0.05, *: *p* < 0.05, **: *p* < 0.01). (e) Three days after tail vein injection, mouse kidneys were paraffin-sectioned and stained with HE (black arrows indicate inflammatory sites under a 10× microscope and red arrows indicate hyphae under a 40× microscope).

In order to further investigate the impact of Spf1 and related ER-PM contacts on *C. albicans* infection, we conducted *in* *vitro* and *in* *vivo* infection experiments. We first constructed an *in* *vitro* Transwell model using bEnd.3 mouse brain endothelial cells to simulate the physical barrier defence against pathogen invasion *in* *vivo*. Meanwhile, Alexa594-labelled concanavalin A (ConA) can selectively bind fungal cell wall mannan and glucan but could not penetrate the host cytoplasmic membrane. Therefore, it is widely used to stain fungal cells not invading the host cells (Imberty et al. 1991; Panchuk-Voloshina et al. 1999). We stained the fungal cells with Alexa594-labelled ConA to evaluate fungal invasion of the host endothelial cells. As shown in Figure 6a, the WT strain led to remarkable disruption of the endothelial cell layer, with most of the fungal cells not staining by Alexa594-labelled ConA. This indicated the WT cells strongly invade into the endothelial cells, leading to endothelial cell damage. In contrast, the four mutants exhibited strong red fluorescence intensity, not resulting in the disruption of the endothelial cell layer. Statistical analysis further showed that the four mutants had much higher ratio of ConA intensity to CFW intensity (Figure 6b), confirming the lower invading capacity of the mutants than that of WT.

Subsequently, we utilised a systemic infection mouse model to evaluate the virulence of the strains. Following the injection of *C. albicans* into the tail vein of mice, we conducted continuous observation and plotted the mouse survival curve (Figure 6c). At the end of day 7, all mice injected with the WT strain died, and at the end of day 8, all mice injected with the *ist2-/-* strain died, whereas the mice infected with the *spf1-/-*, *tcb1-/-tcb3-/-*, and *spf1-/-ist2-/-tcb1-/-tcb3-/-* strains did not experience mortality. Moreover, CFU assays indicated that there was no significant difference in invasive ability between the *ist2-/-* and the WT, both showing severe fungal burdens in the kidneys. In contrast, the mice infected with the *spf1-/-*, *tcb1-/-tcb3-/-*, and *spf1-/-ist2-/-tcb1-/-tcb3-/-* strains had much lighter fungal burdens in the kidneys (Figure 6d). Subsequent paraffin sectioning and HE staining of the mouse kidneys showed that there was almost no inflammation caused by *C. albicans* infection in the kidneys of mice infected with the *spf1-/-*, *tcb1-/-tcb3-/-*, and *spf1-/-ist2-/-tcb1-/-tcb3-/-* strains, whereas pronounced inflammation was observed in the kidneys infected with the WT and *ist2-/-*, accompanied by abundant hyphae present in the tissues under a 40-fold magnification (Figure 6e). Results from *in* *vitro* and *in* *vivo C. albicans* infection experiments suggested that deletion of the *SPF1* gene reduced the infectivity of *C. albicans* by decreasing ER-PM contacts. However, the sole loss of Ist2 leads to a decrease in the secretion level of virulence factors, but its impact on the virulence of *C. albicans* is not significant. The survival rate of the mice injected with the *ist2-/-* strain was significantly lower than that of the mice infected with the *spf1-/-*, *tcb1-/-tcb3-/-*, and *spf1-/-ist2-/-tcb1-/-tcb3-/-* strains. This also indicates that the loss of *IST2* is not sufficient to diminish the virulence of *C. albicans* during systemic infection.

## 4. Discussion

The *SPF1* gene encodes an endoplasmic reticulum P-type ATPase pump, which plays an important role in maintaining endoplasmic reticulum calcium ion balance and regulating intracellular calcium homoeostasis in fungal cells (Li et al. 2018). Calcium ions are not only essential divalent ions for the growth of eukaryotes but also a widely distributed second messenger involved in various developmental signals and specific cellular response pathways. Disruption of fungal intracellular calcium homoeostasis can lead to growth defects and even cell death in all organisms (Liu et al. 2015). Furthermore, the *SPF1* gene has various physiological functions in fungal survival and pathogenesis. Our previous research has shown that the *SPF1* gene is involved in multiple processes of *C. albicans* growth and development. It regulates fungal cell wall synthesis and lipid metabolism, processes closely associated with the recognition and response of fungi to the host immune system (Yu et al. 2012b). Moreover, the imbalance in endoplasmic reticulum calcium homoeostasis resulting from *SPF1* gene deletion further causes a loss of cell wall integrity and defects in hyphal development in *C. albicans* (Yu et al. 2013, 2019).

In this research, we focused on the critical ER-PM contacts in *C. albicans*, exploring the regulatory mechanism of the *SPF1* gene in immune response from a new perspective. Initially, we introduced Ist2 and Tcb1/3 deletion mutants, which are ER-PM tethering proteins, and found that the *SPF1* gene affects the function of these tethering proteins, resulting in reduced ER-PM contact. Additionally, with no significant changes in the expression levels of virulence factors, the reduced levels of these factors in the gene deletion strains also indicate that abnormalities in the secretion process are related to the absence of these tethering proteins. More importantly, these gene-deleted mutants showed a reduced infection capability in both *in vivo* and *in vitro* experiments, demonstrating the role of ER-PM contacts in regulating immune response. Certainly, the infectivity of *C. albicans* is not solely dependent on its ability to colonise the host and release virulence factors that damage the host’s defensive barriers (d’Enfert et al. 2021), in immune responses, the growth ability and cell wall structure of *C. albicans* also play crucial roles. Experimental results from defective strains show that even with the loss of ER-PM contacts while maintaining growth ability and cell wall integrity due to *SPF1* deletion, the strains still exhibited a significant decrease in infectivity and damage. This decline reveals that the reduction in inflammation and immune response upon *SPF1* deletion is a complex issue; the ER-PM contacts may have a parallel and synergistic regulatory relationship with the growth ability or cell wall integrity. Moreover, the ER-PM contacts might influence fungal growth and cell wall synthesis upstream, thereby further modulating the immune response. Since the strength of the immune response in a healthy host partially reflects the severity of the infection, this also corroborates the decreased infectivity and mortality of *C. albicans* in both *in* *vivo* and *in* *vitro* infection experiments.

**Figure 7.**
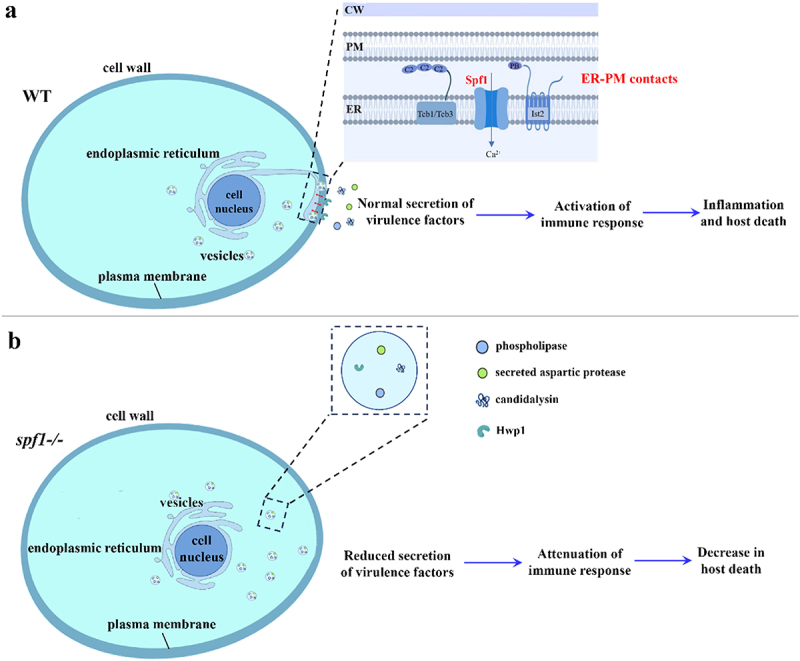
Schematic diagram of *Candida albicans* virulence factor secretion controlled by Spf1-maintained ER-PM contacts. (a) Spf11-governed ER-PM contacts for regulation of virulence factor secretion and immune response in the WT strain. (b) Defect in the formation of ER-PM contacts in the *spf1-/-* strain with reduced secretion of virulence factors and attenuation of the immune response.

The classical endoplasmic reticulum-Golgi-plasma membrane secretion pathway is the main secretion pathway for most proteins. Gene sequences show that proteins such as Hwp1, SAP, and others contain signal peptide sequences related to this pathway (Chaffin et al. 1998). Candidalysin must also undergo further cleavage and processing in the secretion pathway to form mature candidalysin (Russell et al. 2022). Additionally, all five phospholipase genes in *C. albicans* were found to contain secretion signal peptide sequences (Sorgo et al. 2013). This suggests that the protein secretion pathway plays a crucial role in the secretion of virulence factors in *C. albicans*. The ER-PM contact is also a critical pathway from the endoplasmic reticulum to the plasma membrane. The plasma membrane absorbs and transports large amounts of proteins and lipids through vesicles, playing an important role in secretion pathways and endocytosis. Therefore, the endoplasmic reticulum needs to communicate with the plasma membrane promptly, regulate biosynthesis, and adapt to the reshaping of the plasma membrane after changes in internal and external environments (Manford et al. 2012). Experimental results show a direct correlation between the secretion of certain virulence factors in *C. albicans* and Spf1-maintained ER-PM contact. In the WT strain, the existence of the ER-PM contacts leads to a shortened distance from the endoplasmic reticulum to the plasma membrane, allowing the endoplasmic reticulum to better receive membrane component information returned from the plasma membrane, thereby stabilising secretion in the ER-PM secretion pathway. The normal secretion of virulence factors further activates the immune response, leading to severe tissue inflammation and host death (Figure 7a). However, in the *SPF1*-deficient strain, the decrease in contacts caused by the abnormal function of ER-PM tethering proteins affects this stable and normal secretion process, leading to the attenuation of immune response and the decrease in host death (Figure 7b).

In conclusion, this study revealed that the *SPF1* gene regulates the immune response of the host during systemic *C. albicans* infection by regulating the function of ER-PM tethering proteins, the deletion of *SPF1* reduced the formation of ER-PM contacts, leading to the attenuation of virulence factor secretion, and ultimately affecting the virulence of *C. albicans*. This discovery provides new clues for further elucidating the relationship between pathogenic fungi and the immune system, and also provides a theoretical basis for therapeutic strategies against fungal infections.

## Supplementary Material

Figure S2 yeast gfp.jpg

Figure S1 expression tnf.jpg

Figure S3.jpg

Supplemental material.docx

## References

[cit0001] Arita GS, Faria DR, Capoci IRG, Kioshima ES, Bonfim-Mendonça PS, Svidzinski TIE. 2022. Cell wall associated proteins involved in filamentation with impact on the virulence of *Candida albicans*. Microbiol Res. 258:126996. doi: 10.1016/j.micres.2022.126996. 35247799

[cit0002] Bai FY. 2014. Association of genotypes with infection types and antifungal susceptibilities in *Candida albicans* as revealed by recent molecular typing strategies. Mycology. 5(1):1–9. doi: 10.1080/21501203.2014.899525. 24772369 PMC3979442

[cit0003] Balish E, Wagner RD, Vázquez-Torres A, Pierson C, Warner T. 1998. Candidiasis in interferon-γ knockout (IFN-γ-/-) mice. J Infect Dis. 178(2):478–487. doi: 10.1086/515645. 9697730

[cit0004] Bojang E, Ghuman H, Kumwenda P, Hall RA. 2021. Immune sensing of *Candida albicans*. J Fungi. 7(2):119. doi: 10.3390/jof7020119. PMC7914548 33562068

[cit0005] Brand A, MacCallum DM, Brown AJP, Gow NAR, Odds FC. 2004. Ectopic expression of URA3 can influence the virulence phenotypes and proteome of *Candida albicans* but can be overcome by targeted reintegration of URA3 at the RPS10 locus. Eukaryot Cell. 3(4):900–909. doi: 10.1128/EC.3.4.900-909.2004. 15302823 PMC500875

[cit0006] Chaffin WL, López-Ribot JL, Casanova M, Gozalbo D, Martínez JP. 1998. Cell wall and secreted proteins of *Candida albicans*: identification, function, and expression. Microbiol Mol Biol Rev. 62(1):130–180. doi: 10.1128/MMBR.62.1.130-180.1998. 9529890 PMC98909

[cit0007] Chang CL, Chen YJ, Liou J. 2017. Er-plasma membrane junctions: why and how do we study them? BBA-Molar Cell Res. 1864(9):1494–1506. doi: 10.1016/j.bbamcr.2017.05.018. PMC5542405 28554772

[cit0008] Charbonneau H, Tonks NK, Walsh KA, Fischer EH. 1988. The leukocyte common antigen (CD45): a putative receptor-linked protein tyrosine phosphatase. Proc Natl Acad Sci USA. 85(19):7182–7186. doi: 10.1073/pnas.85.19.7182. 2845400 PMC282148

[cit0009] Costa CR, Jesuíno RSA, de Aquino Lemos J, de Fátima Lisboa Fernandes O, Hasimoto e Souza LK, Passos XS, Do Rosário Rodrigues Silva M. 2010. Effects of antifungal agents in sap activity of *Candida albicans* isolates. Mycopathologia. 169(2):91–98. doi: 10.1007/s11046-009-9232-6. 19685156

[cit0010] Cronin SR, Rao R, Hampton RY. 2002. Cod1p/Spf1p is a P-type ATPase involved in ER function and Ca^2+^ homeostasis. J Cell Biol. 157(6):1017–1028. doi: 10.1083/jcb.200203052. 12058017 PMC2174042

[cit0011] Daley JM, Thomay AA, Connolly MD, Reichner JS, Albina JE. 2008. Use of Ly6G-specific monoclonal antibody to deplete neutrophils in mice. J Leukocyte Biol. 83(1):64–70. doi: 10.1189/jlb.0407247. 17884993

[cit0012] d’Enfert C, Kaune AK, Alaban LR, Chakraborty S, Cole N, Delavy M, Kosmala D, Marsaux B, Fróis-Martins R, Morelli M, et al. 2021. The impact of the fungus-host-microbiota interplay upon *Candida albicans* infections: current knowledge and new perspectives. FEMS Microbiol Rev. 45(3):fuaa060. doi: 10.1093/femsre/fuaa060. 33232448 PMC8100220

[cit0013] Dixon RE, Trimmer JS. 2023. Endoplasmic reticulum-plasma membrane junctions as sites of depolarization-induced Ca^2+^ signaling in excitable cells. Annu Rev Physiol. 85(1):217–243. doi: 10.1146/annurev-physiol-032122-104610. 36202100 PMC9918718

[cit0014] Duddy ME, Alter A, Bar-Or A. 2004. Distinct profiles of human B cell effector cytokines: a role in immune regulation? J Immunol. 172(6):3422–3427. doi: 10.4049/jimmunol.172.6.3422. 15004141

[cit0015] Fan Y, He H, Dong Y, Pan H. 2013. Hyphae-specific genes HGC1, ALS3, HWP1, and ECE1 and relevant signaling pathways in *Candida albicans*. Mycopathologia. 176(5–6):329–335. doi: 10.1007/s11046-013-9684-6. 24002103

[cit0016] Ferro MP, Heilshorn SC, Owens RM. 2020. Materials for blood brain barrier modeling *in vitro*. Mater Sci Eng. 140:100522. doi: 10.1016/j.mser.2019.100522. PMC7864217 33551572

[cit0017] Foley JGD, Bard JBL. 2002. Apoptosis in the cortex of the developing mouse kidney. J Anat. 201(6):477–484. doi: 10.1046/j.1469-7580.2002.00114.x. 12489759 PMC1570997

[cit0018] Fonzi WA. 2009. The protein secretory pathway of *Candida albicans*. Mycoses. 52(4):291–303. doi: 10.1111/j.1439-0507.2008.01673.x. 19207839

[cit0019] Förster C, Kane PM. 2000. Cytosolic Ca^2+^ homeostasis is a constitutive function of the V-ATPase in *Saccharomyces cerevisiae*. J Biol Chem. 275(49):38245–38253. doi: 10.1074/jbc.M006650200. 10991947

[cit0020] Griffiths JS, Camilli G, Kotowicz NK, Ho J, Richardson JP, Naglik JR. 2021. Role for IL-1 family cytokines in fungal infections. Front Microbiol. 12:633047. doi: 10.3389/fmicb.2021.633047. 33643264 PMC7902786

[cit0021] Gullotta GS, De Feo D, Friebel E, Semerano A, Scotti GM, Bergamaschi A, Butti E, Brambilla E, Genchi A, Capotondo A, et al. 2023. Age-induced alterations of granulopoiesis generate atypical neutrophils that aggravate stroke pathology. Nat Immunol. 24(6):925–940. doi: 10.1038/s41590-023-01505-1. 37188941

[cit0022] Harris DP, Haynes L, Sayles PC, Duso DK, Eaton SM, Lepak NM, Johnson LL, Swain SL, Lund FE. 2000. Reciprocal regulation of polarized cytokine production by effector B and T cells. Nat Immunol. 1(6):475–482. doi: 10.1038/82717. 11101868

[cit0023] Hasenberg A, Hasenberg M, Männ L, Neumann F, Borkenstein L, Stecher M, Kraus A, Engel DR, Klingberg A, Seddigh P, et al. 2015. Catchup: a mouse model for imaging-based tracking and modulation of neutrophil granulocytes. Nat Methods. 12(5):445–452. doi: 10.1038/nmeth.3322. 25775045

[cit0024] Herring AC, Falkowski NR, Chen GH, McDonald RA, Toews GB, Huffnagle GB. 2005. Transient neutralization of tumor necrosis factor alpha can produce a chronic fungal infection in an immunocompetent host: potential role of immature dendritic cells. Infect Immun. 73(1):39–49. doi: 10.1128/IAI.73.1.39-49.2005. 15618139 PMC538928

[cit0025] Hu QL, Zhong H, Wang XR, Han L, Ma SS, Li L, Wang Y. 2024. Mitochondrial phosphate carrier plays an important role in virulence of *Candida albicans*. Mycology. 1–13. doi: 10.1080/21501203.2024.2354876. PMC11899212 40083413

[cit0026] Hube B, Sanglard D, Odds FC, Hess D, Monod M, Schäfer W, Brown AJ, Gow NA. 1997. Disruption of each of the secreted aspartyl proteinase genes *SAP1*, *SAP2*, and *SAP3* of *Candida albicans* attenuates virulence. Infect Immun. 65(9):3529–3538. doi: 10.1128/iai.65.9.3529-3538.1997. 9284116 PMC175503

[cit0027] Imberty A, Hardman KD, Carver JP, Perez S. 1991. Molecular modelling of protein-carbohydrate interactions. Docking of monosaccharides in the binding site of concanavalin A. Glycobiology. 1(6):631–642. doi: 10.1093/glycob/1.6.631. 1822243

[cit0028] Jawale CV, Biswas PS. 2021. Local antifungal immunity in the kidney in disseminated candidiasis. Curr Opin Microbiol. 62:1–7. doi: 10.1016/j.mib.2021.04.005. 33991758 PMC8286321

[cit0029] Jiang L, Xu D, Hameed A, Fang T, Bakr Ahmad Fazili A, Asghar F. 2018. The plasma membrane protein Rch1 and the Golgi/ER calcium pump Pmr1 have an additive effect on filamentation in *Candida albicans*. Fungal Genet Biol. 115:1–8. doi: 10.1016/j.fgb.2018.04.001. 29621626

[cit0030] Keegan AD, Leonard WJ, Zhu J. 2021. Recent advances in understanding the role of IL-4 signaling. Fac Rev. 10:71. doi: 10.12703/r/10-71. 34557875 PMC8442009

[cit0031] Kulkarni U, Karsten CM, Kohler T, Hammerschmidt S, Bommert K, Tiburzy B, Meng L, Thieme L, Recke A, Ludwig RJ, et al. 2016. IL-10 mediates plasmacytosis-associated immunodeficiency by inhibiting complement-mediated neutrophil migration. J Allergy Clin Immun. 137(5):1487–1497.e6. doi: 10.1016/j.jaci.2015.10.018. 26653800

[cit0032] Li C, Qian T, He R, Wan C, Liu Y, Yu H. 2021. Endoplasmic reticulum-plasma membrane contact sites: regulators, mechanisms, and physiological functions. Front Cell Dev Biol. 9:627700. doi: 10.3389/fcell.2021.627700. 33614657 PMC7889955

[cit0033] Li XV, Leonardi I, Putzel GG, Semon A, Fiers WD, Kusakabe T, Lin WY, Gao IH, Doron I, Gutierrez-Guerrero A, et al. 2022. Immune regulation by fungal strain diversity in inflammatory bowel disease. Nature. 603(7902):672–678. doi: 10.1038/s41586-022-04502-w. 35296857 PMC9166917

[cit0034] Li Y, Sun L, Lu C, Gong Y, Li M, Sun S. 2018. Promising antifungal targets against *Candida albicans* based on Ion Homeostasis. Front Cell Infect Microbiol. 8:286. doi: 10.3389/fcimb.2018.00286. 30234023 PMC6131588

[cit0035] Liang C, Zhang B, Cui L, Li J, Yu Q, Li M. 2018. Mgm1 is required for maintenance of mitochondrial function and virulence in *Candida albicans*. Fungal Genet Biol. 120:42–52. doi: 10.1016/j.fgb.2018.09.006. 30240789

[cit0036] Liu S, Hou Y, Liu W, Lu C, Wang W, Sun S. 2015. Components of the Calcium-Calcineurin signaling pathway in fungal cells and their potential as antifungal targets. Eukaryot Cell. 14(4):324–334. doi: 10.1128/EC.00271-14. 25636321 PMC4385803

[cit0037] Lodyga M, Hinz B. 2020. TGF-β1 – a truly transforming growth factor in fibrosis and immunity. Semin Cell Dev Biol. 101:123–139. doi: 10.1016/j.semcdb.2019.12.010. 31879265

[cit0038] Luna-Tapia A, DeJarnette C, Sansevere E, Reitler P, Butts A, Hevener KE, Palmer GE, Mitchell AP. 2019. The vacuolar Ca^2+^ ATPase pump pmc1p is required for *Candida albicans* pathogenesis. mSphere. 4(1):e00715–18. doi: 10.1128/mSphere.00715-18. PMC6365616 30728284

[cit0039] Manford AG, Stefan CJ, Yuan HL, MacGurn JA, Emr SD. 2012. ER-to-plasma membrane tethering proteins regulate cell signaling and ER morphology. Develop Cell. 23(6):1129–1140. doi: 10.1016/j.devcel.2012.11.004. 23237950

[cit0040] Maras B, Maggiore A, Mignogna G, D’Erme M, Angiolella L, Ahmad A. 2021. Hyperexpression of CDRs and HWP1 genes negatively impacts on *Candida albicans* virulence. PLOS One. 16(6):e0252555. doi: 10.1371/journal.pone.0252555. 34061886 PMC8168907

[cit0041] Moyes DL, Wilson D, Richardson JP, Mogavero S, Tang SX, Wernecke J, Höfs S, Gratacap RL, Robbins J, Runglall M, et al. 2016. Candidalysin is a fungal peptide toxin critical for mucosal infection. Nature. 532(7597):64–68. doi: 10.1038/nature17625. 27027296 PMC4851236

[cit0042] Nailis H, Coenye T, Van Nieuwerburgh F, Deforce D, Nelis HJ. 2006. Development and evaluation of different normalization strategies for gene expression studies in *Candida albicans* biofilms by real-time PCR. BMC Mol Biol. 7(1):25. doi: 10.1186/1471-2199-7-25. 16889665 PMC1557526

[cit0043] Nciki S, Oderinlo OO, Gulube Z, Osamudiamen PM, Idahosa KC, Patel M. 2020. Mezoneuron benthamianum inhibits cell adherence, hyphae formation, and phospholipase production in *Candida albicans*. Arch Microbiol. 202(9):2533–2542. doi: 10.1007/s00203-020-01972-2. 32656677

[cit0044] Niewerth M, Korting HC. 2001. Phospholipases of *Candida albicans*. Mycoses. 44(9–10):361–367. doi: 10.1046/j.1439-0507.2001.00685.x. 11766099

[cit0045] Panchuk-Voloshina N, Haugland Rosaria P, Bishop-Stewart J, Bhalgat MK, Millard PJ, Mao F, Leung WY, Haugland Richard P. 1999. Alexa Dyes, a series of new fluorescent dyes that yield exceptionally bright, photostable conjugates. J Histochem Cytochem. 47(9):1179–1188. doi: 10.1177/002215549904700910. 10449539

[cit0046] Pillay J, Tak T, Kamp VM, Koenderman L. 2013. Immune suppression by neutrophils and granulocytic myeloid-derived suppressor cells: similarities and differences. Cell Mol Life Sci. 70(20):3813–3827. doi: 10.1007/s00018-013-1286-4. 23423530 PMC3781313

[cit0047] Price MF, Wilkinson ID, Gentry LO. 1982. Plate method for detection of phospholipase activity in *Candida albicans*. Med Mycol. 20(1):7–14. doi: 10.1080/00362178285380031. 7038928

[cit0048] Rabanel JM, Piec PA, Landri S, Patten SA, Ramassamy C. 2020. Transport of PEGylated-pla nanoparticles across a blood brain barrier model, entry into neuronal cells and *in* *vivo* brain bioavailability. J Control Release. 328:679–695. doi: 10.1016/j.jconrel.2020.09.042. 32979453

[cit0049] Richardson JP, Moyes DL. 2015. Adaptive immune responses to *Candida albicans* infection. Virulence. 6(4):327–337. doi: 10.1080/21505594.2015.1004977. 25607781 PMC4601188

[cit0050] Russell CM, Schaefer KG, Dixson A, Gray AL, Pyron RJ, Alves DS, Moore N, Conley EA, Schuck RJ, White TA, et al. 2022. The *Candida albicans* virulence factor candidalysin polymerizes in solution to form membrane pores and damage epithelial cells. eLife. 11:e75490. doi: 10.7554/eLife.75490. 36173096 PMC9522247

[cit0051] Schild L, Heyken A, De Groot PWJ, Hiller E, Mock M, De Koster C, Horn U, Rupp S, Hube B. 2011. Proteolytic cleavage of covalently linked cell wall proteins by *Candida albicans* Sap9 and Sap10. Eukaryot Cell. 10(1):98–109. doi: 10.1128/EC.00210-10. 21097664 PMC3019796

[cit0052] Schulz TA, Creutz CE. 2004. The tricalbin C2 domains: lipid-binding properties of a novel, synaptotagmin-like yeast protein family. Biochemistry. 43(13):3987–3995. doi: 10.1021/bi036082w. 15049706

[cit0053] Sorgo AG, Heilmann CJ, Brul S, de Koster CG, Klis FM. 2013. Beyond the wall: *Candida albicans* secret(e)s to survive. FEMS Microbiol Lett. 338(1):10–17. doi: 10.1111/1574-6968.12049. 23170918

[cit0054] Stefan CJ, Manford AG, Emr SD. 2013. ER-PM connections: sites of information transfer and inter-organelle communication. Curr Opin Cell Biol. 25(4):434–442. doi: 10.1016/j.ceb.2013.02.020. 23522446 PMC4074705

[cit0055] Talapko J, Juzbašić M, Matijević T, Pustijanac E, Bekić S, Kotris I, Škrlec I. 2021. *Candida albicans*—the virulence factors and clinical manifestations of infection. J Fungi. 7(2):79. doi: 10.3390/jof7020079. PMC7912069 33499276

[cit0056] Trinchieri G, Perussia B. 1985. Immune interferon: a pleiotropic lymphokine with multiple effects. Immunol Today. 6(4):131–136. doi: 10.1016/0167-5699(85)90080-5. 25289500

[cit0057] Walther A, Wendland J. 2003. An improved transformation protocol for the human fungal pathogen *Candida albicans*. Curr Genet. 42(6):339–343. doi: 10.1007/s00294-002-0349-0. 12612807

[cit0058] Wegener J, Seebach J. 2014. Experimental tools to monitor the dynamics of endothelial barrier function: a survey of *in* *vitro* approaches. Cell Tissue Res. 355(3):485–514. doi: 10.1007/s00441-014-1810-3. 24585359

[cit0059] Winer J, Jung CKS, Shackel I, Williams PM. 1999. Development and validation of real-time quantitative reverse transcriptase–polymerase chain reaction for monitoring gene expression in cardiac myocytes *in* *vitro*. Anal Biochem. 270(1):41–49. doi: 10.1006/abio.1999.4085. 10328763

[cit0060] Wolf W, Kilic A, Schrul B, Lorenz H, Schwappach B, Seedorf M, Beh C. 2012. Yeast Ist2 recruits the endoplasmic reticulum to the plasma membrane and creates a ribosome-free membrane microcompartment. PLOS One. 7(7):e39703. doi: 10.1371/journal.pone.0039703. 22808051 PMC3392263

[cit0061] Wong AKO, Young BP, Loewen CJR. 2021. Ist2 recruits the lipid transporters Osh6/7 to ER-PM contacts to maintain phospholipid metabolism. J Cell Biol. 220(9):e201910161. doi: 10.1083/jcb.201910161. 34259806 PMC8282664

[cit0062] Yang L, Zhu H, Li M, Yu Q, Krysan DJ. 2022. The tricalbin-family endoplasmic reticulum-plasma membrane tethering proteins attenuate ros-involved caspofungin sensitivity in *Candida albicans*. Microbiol Spectr. 10(6):e02079-22. doi: 10.1128/spectrum.02079-22. 36445092 PMC9769562

[cit0063] Yu Q, Ding X, Bing Z, Xu N, Cheng X, Qian K, Biao Z, Xing L, Li M. 2013. The P-type ATPase Spf1 is required for endoplasmic reticulum functions and cell wall integrity in *Candida albicans*. Int J Med Microbiol. 303(5):257–266. doi: 10.1016/j.ijmm.2013.05.003. 23731904

[cit0064] Yu Q, Ding X, Bing Z, Xu N, Jia C, Mao J, Biao Z, Xing L, Li M. 2014. Inhibitory effect of verapamil on *Candida albicans* hyphal development, adhesion and gastrointestinal colonization. FEMS Yeast Res. 14(4):633–641. doi: 10.1111/1567-1364.12150. 24650198

[cit0065] Yu Q, Ma T, Ma C, Zhang B, Li M. 2019. Multifunction of the ER P-type calcium pump Spf1 during hyphal development in *Candida albicans*. Mycopathologia. 184(5):573–583. doi: 10.1007/s11046-019-00372-5. 31473908

[cit0066] Yu Q, Wang H, Cheng X, Xu N, Ding X, Xing L, Li M. 2012a. Roles of Cch1 and Mid1 in morphogenesis, oxidative stress response and virulence in *Candida albicans*. Mycopathologia. 174(5–6):359–369. doi: 10.1007/s11046-012-9569-0. 22886468

[cit0067] Yu Q, Wang H, Xu N, Cheng X, Wang Y, Zhang B, Xing L, Li M. 2012b. Spf1 strongly influences calcium homeostasis, hyphal development, biofilm formation and virulence in *Candida albicans*. Microbiology. 158(9):2272–2282. doi: 10.1099/mic.0.057232-0. 22745267

