# Supplementary figures and images for: The P-type calcium pump Spf1 regulates immune response by maintenance of the endoplasmic reticulum-plasma membrane contacts during Candida albicans systemic infection
Source: Mycology. 2024 Nov 7;16(2):856–75. doi: 10.1080/21501203.2024.2409299 (PMC12096691; doi:10.1080/21501203.2024.2409299)

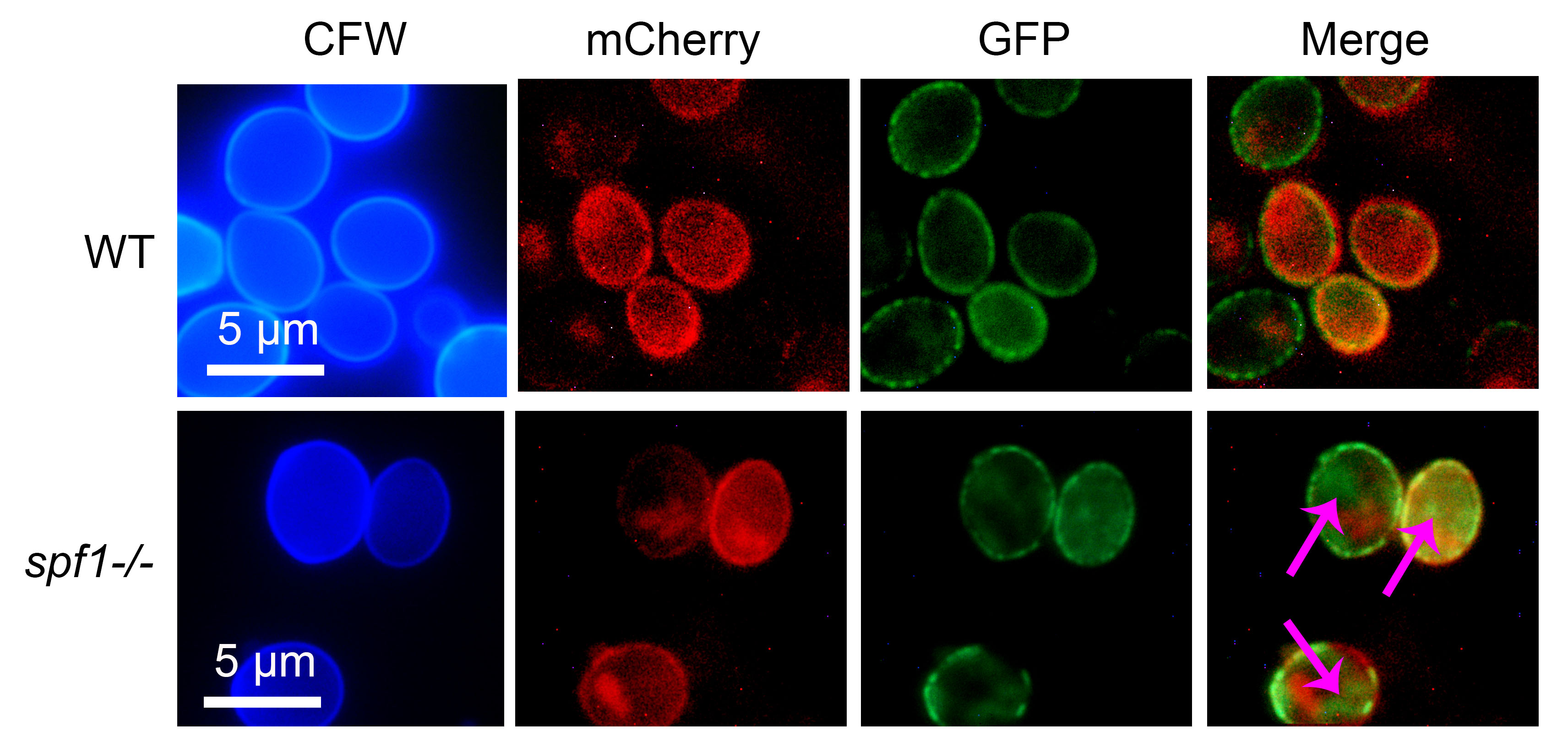

Supplement: Figure S2 yeast gfp.jpg [file TMYC_A_2409299_SM4657.jpg]

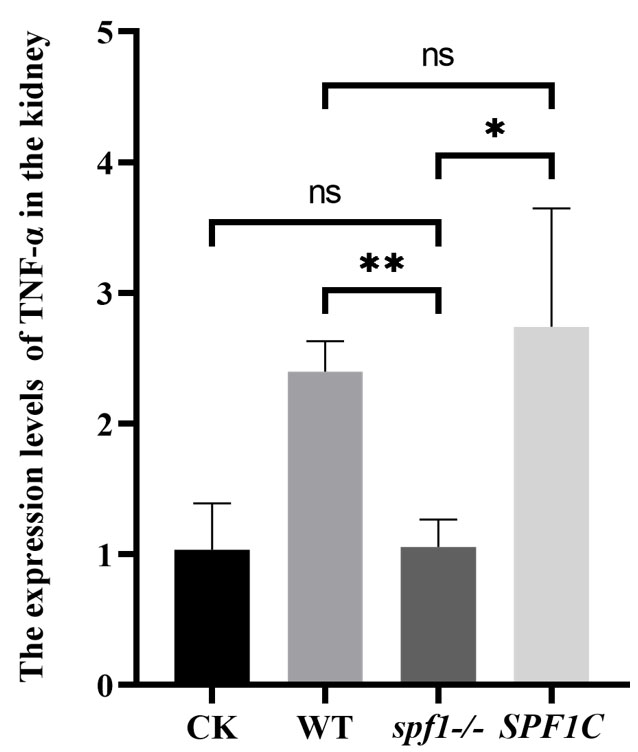

Supplement: Figure S1 expression tnf.jpg [file TMYC_A_2409299_SM4656.jpg]

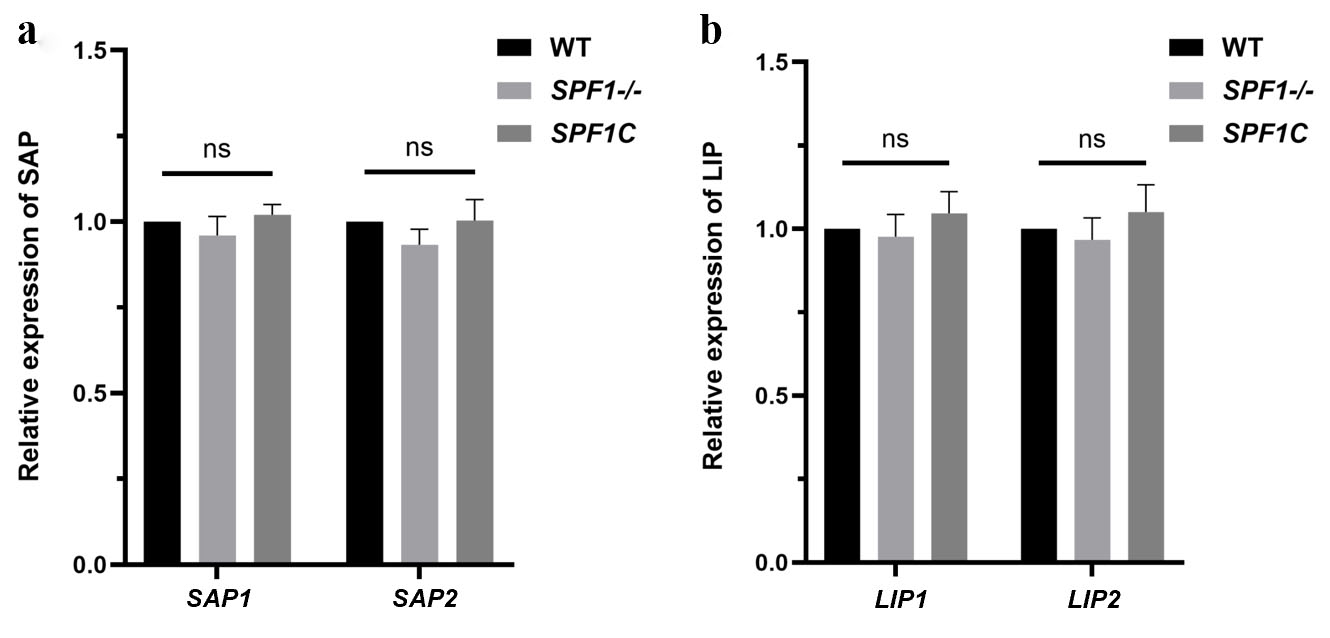

Supplement: Figure S3.jpg [file TMYC_A_2409299_SM4655.jpg]
