# Supplementary material for: The P-type calcium pump Spf1 regulates immune response by maintenance of the endoplasmic reticulum-plasma membrane contacts during Candida albicans systemic infection
Source: Mycology. 2024 Nov 7;16(2):856–75. doi: 10.1080/21501203.2024.2409299 (PMC12096691; doi:10.1080/21501203.2024.2409299)
Supplement: Supplemental material.docx [file TMYC_A_2409299_SM4654.docx]

**Supporting Information**

**The P-type calcium pump Spf1 regulates immune response by maintenance of the endoplasmic reticulum-plasma membrane contacts during *Candida albicans* systemic infection**

Ji Yuchao, Chen Dou, Shao Menglin, Liu Zhuo, Mingchun Li*, Yu Qilin *

National Key Laboratory of Intelligent Tracking and Forecasting for Infectious Diseases, College of Life Sciences, Nankai University, Tianjin, China, 300071

E-mail: yuqilin@mail.nankai.edu.cn (Y.Q.); nklimingchun@163.com (L.M.)

**Table S1 The *C. albicans* strains used in the study**

| Name | Brief Description |
| --- | --- |
| WT (BWP17) | *ura3Δ::λimm434/ura3Δ::λimm434 his1::hisG/his1::hisG arg4::hisG/arg4::hisG* |
| *ist2-/-* | *ura3Δ::λimm434/ura3Δ::λimm434 his1::hisG/his1::hisG arg4::hisG/arg4::hisG ist2::ARG4/ist2::dpl200* |
| *spf1-/-* | *ura3Δ::λimm434/ura3Δ::λimm434 his1::hisG/his1::hisG arg4::hisG/arg4::hisG spf1::ARG4/spf1::dpl200* |
| *tcb1-/- tcb3-/-* | *ura3Δ::λimm434/ura3Δ::λimm434 his1::hisG/his1::hisG arg4::hisG/arg4::hisG tcb3::ARG4/tcb3::dpl200, tcb1::dpl200/tcb1::dpl200* |
| *ist2-/-spf1-/-tcb1-/- tcb3-/-* | *ura3Δ::λimm434/ura3Δ::λimm434 his1::hisG/his1::hisG arg4::hisG/arg4::hisG ist2::ARG4/ist2::dpl200，spf1::dp1200/spf1::dpl200，tcb3::dp1200/tcb3::dpl200, tcb1::dpl200/tcb1::dpl200* |
| WT^a^ | *URA3/ura3Δ::λimm434 his1::hisG/his1::hisG arg4::hisG/arg4::hisG* |
| *ist2-/-*^a^ | *URA3/ura3Δ::λimm434 his1::hisG/his1::hisG arg4::hisG/arg4::hisG ist2::ARG4/ist2::dpl200* |
| *spf1-/-*^a^ | *URA3/ura3Δ::λimm434 his1::hisG/his1::hisG arg4::hisG/arg4::hisG spf1::ARG4/spf1::dpl200* |
| *tcb1-/- tcb3-/-*^a^ | *URA3/ura3Δ::λimm434 his1::hisG/his1::hisG arg4::hisG/arg4::hisG tcb3::ARG4/tcb3::dpl200, tcb1::dpl200/tcb1::dpl200* |
| *ist2-/-spf1-/-tcb1-/- tcb3-/-*^a^ | *URA3/ura3Δ::λimm434 his1::hisG/his1::hisG arg4::hisG/arg4::hisG ist2::ARG4/ist2::dpl200，spf1::dp1200/spf1::dpl200，tcb3::dp1200/tcb3::dpl200, tcb1::dpl200/tcb1::dpl200* |
| WT-Hwp1-mCherry | *ura3Δ::λimm434/ura3Δ::λimm434 his1::hisG/his1::hisG arg4::hisG/arg4::hisG his1::*pDDB78*-Hwp1-mCherry* |
| *ist2-/-*-Hwp1-mCherry | *ura3Δ::λimm434/ura3Δ::λimm434 his1::hisG/his1::hisG arg4::hisG/arg4::hisG his1::*pDDB78*-Hwp1-mCherry* |
| *spf1-/-*-Hwp1-mCherry | *ura3Δ::λimm434/ura3Δ::λimm434 his1::hisG/his1::hisG arg4::hisG/arg4::hisG his1::*pDDB78*-Hwp1-mCherry* |
| *tcb1-/-tcb3-/-*-Hwp1-mCherry | *ura3Δ::λimm434/ura3Δ::λimm434 his1::hisG/his1::hisG arg4::hisG/arg4::hisG his1::*pDDB78*-Hwp1-mCherry* |
| i*st2-/-spf1-/-tcb1-/- tcb3-/-* -Hwp1-mCherry | *ura3Δ::λimm434/ura3Δ::λimm434 his1::hisG/his1::hisG arg4::hisG/arg4::hisG his1::*pDDB78*-Hwp1-mCherry* |
| *SPF1c* | *ura3Δ::λimm434/ura3Δ::λimm434 his1::hisG/his1::hisG arg4::hisG/arg4::hisG spf1::ARG4/spf1::URA3-dpl200,SPF1,HIS1* |

**Table S2 The plasmids used in the study**

| Name | Brief Description |
| --- | --- |
| pLUBP | Contains the 4.9 kb full-length *IRO1-URA3* fragment, Amp^R^ |
| pGFP-Ist2 | Containing the GFP-Ist2 expressing fragment, *URA3*, Amp^R^ |
| pPH3-mCherry | Containing the PH3-mCherry expressing fragment, *HIS1*, Amp^R^ |
| pHwp1-mCherry | Contains the Hwp1-mCherry fusion fragment, *HIS1*, Amp^R^ |

**Table S3 Information of the primer used in the study**

| Name | Forward Primer（5’-3’） | Reverse Primer（5’-3’） |
| --- | --- | --- |
| URA3 | CGCGGGATTTGGATGGTAT | TCTTGGCTCTTGGTTGGTG |
| Actin | CATTGCTGACAGGATGCAGAAGG | TGCTGGAAGGTGGACAGTGAGG |
| IL-1β | TGGACCTTCCAGGATGAGGACA | GTTCATCTCGGAGCCTGTAGTG |
| IL-4 | ATCATCGGCATTTTGAACGAGGTC | ACCTTGGAAGCCCTACAGACGA |
| IL-6 | TACCACTTCACAAGTCGGAGGC | CTGCAAGTGCATCATCGTTGTTC |
| IL-10 | CGGGAAGACAATAACTGCACCC | CGGTTAGCAGTATGTTGTCCAGC |
| INF-γ | CAGCAACAGCAAGGCGAAAAAGG | TTTCCGCTTCCTGAGGCTGGAT |
| TNF-α | GGTGCCTATGTCTCAGCCTCTT | GCCATAGAACTGATGAGAGGGA |
| TGF-β1 | TGATACGCCTGAGTGGCTGTCT | CACAAGAGCAGTGAGCGCTGAA |


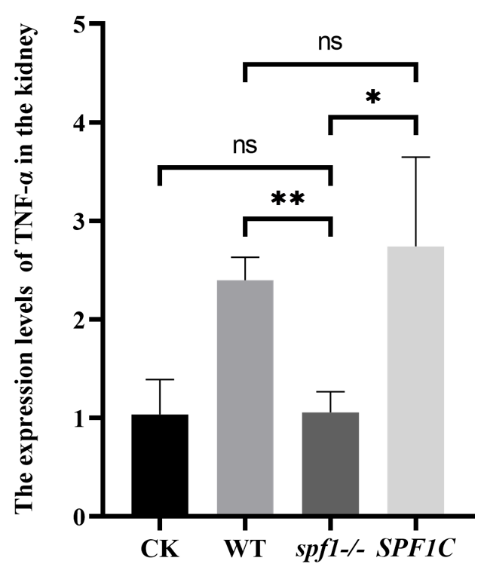


**Figure S1. Expression of TNF-α in the kidneys during infection with different genotypes of *C. albicans.*** The letters “ns” indicate no significant difference between the groups, while the asterisks (*) indicate significant difference (P <0.05).


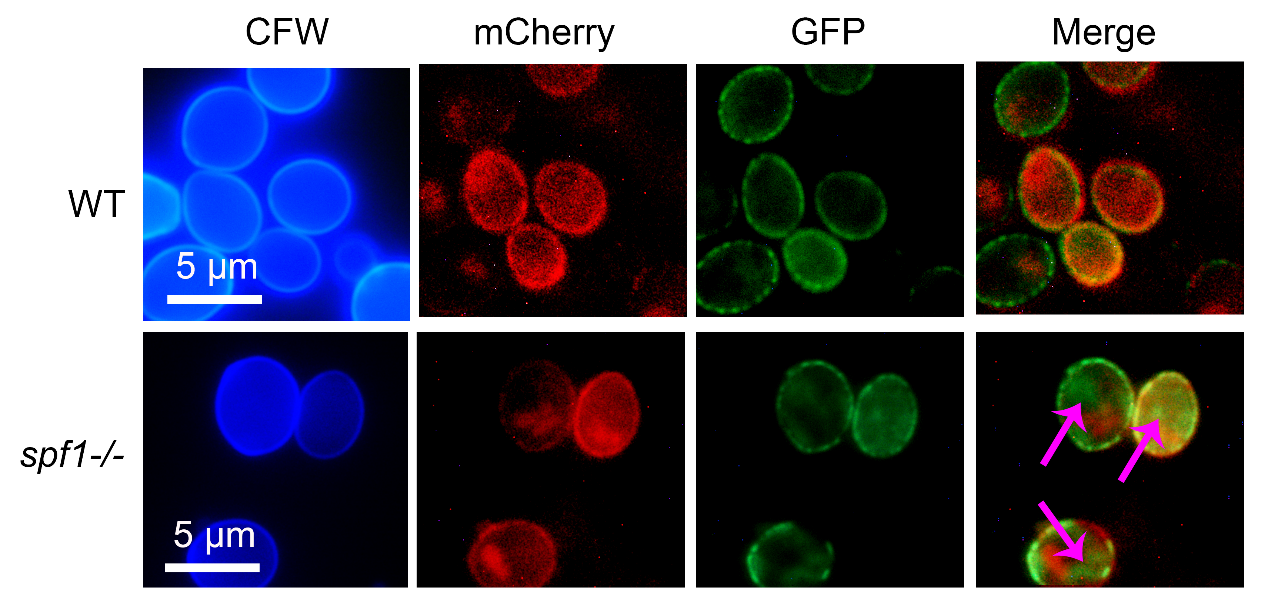


**Figure S2. Localization of GFP-Ist2 in the yeast cells of WT and spf1-/-.** The cells expressing GFP-Ist2 and PH3-mCherry (indicating PM) were cultured in YPD medium for 4 h, followed by staining by CFW for confocal microscopy.


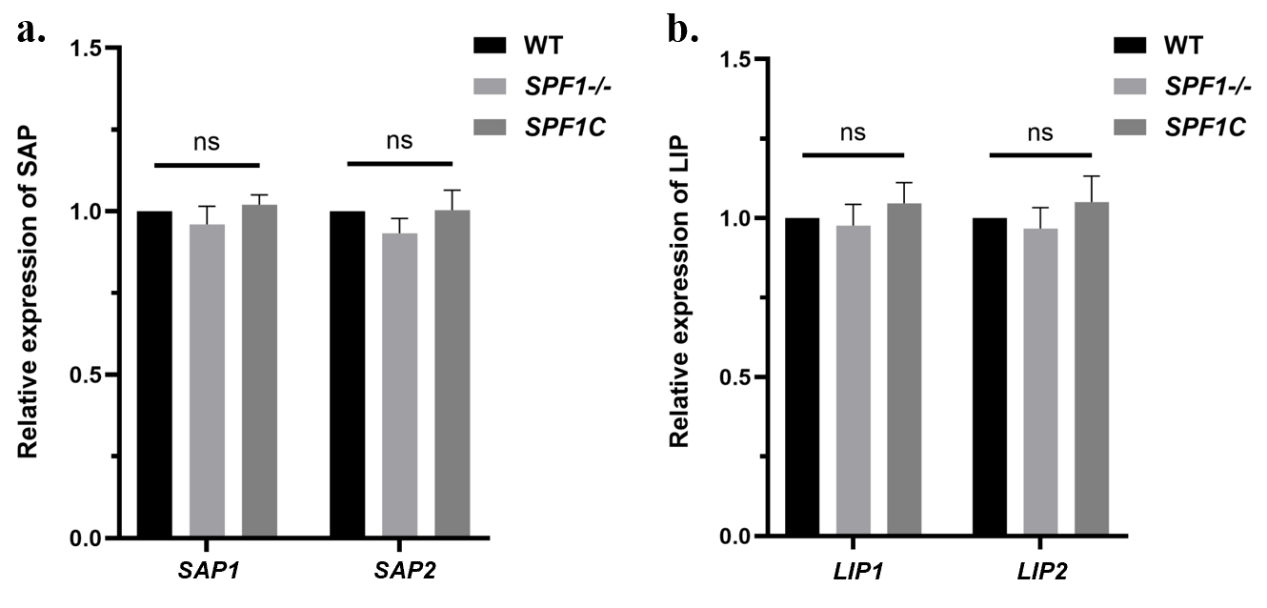


**Figure S3. Expression of *SAP1* and *SAP2* (a), together with *LIP1* and *LIP2* (b), in the hyphal cells of WT, *spf1-/-* and *SPF1c*.** The cells were cultured in RPMI-1640 medium at 37 °C for 4 h, followed by RNA extraction, cDNA preparation and qPCR detection. The letters “ns” indicate no significant difference between the groups (P <0.05).
